# Disease Progression in Multiple System Atrophy: The ASPIRE Multi‐Modal Biomarker Study

**DOI:** 10.1002/ana.70028

**Published:** 2025-08-26

**Authors:** Margherita Fabbri, Natalia del Campo, Wassilios G. Meissner, Vanessa Rousseau, Agnès Sommet, Pierre Payoux, Pierre Gantet, Amel Drif, Hélène Catalá, Claire Thalamas, Christine Tranchant, Franck Durif, Ana Marques, Alexandre Eusebio, Luc Defebvre, Jean‐Christophe Corvol, Stéphane Thobois, Anthime Flaus, Anne‐Gaelle Corbille, Solène Frismand, Beverley Patterson, Alexandra Foubert‐Samier, Anne Pavy‐Le Traon, Germain Arribarat, Patrice Péran, Olivier Rascol, the ASPIRE Study Group

**Affiliations:** ^1^ Clinical Investigation Center CIC1436, Department of Clinical Pharmacology and Neurosciences, Parkinson Expert Centre and NeuroToul Center of Excellence in Neurodegeneration (COEN) of Toulouse; INSERM University of Toulouse, CHU of Toulouse Toulouse France; ^2^ MSA French Reference Center University Hospital Toulouse Toulouse France; ^3^ ToNIC, Toulouse NeuroImaging Center, Université de Toulouse, Inserm, UPS Toulouse France; ^4^ CHU Bordeaux, Service de Neurologie des Maladies Neurodégénératives, CRMR AMS, IMNc Bordeaux France; ^5^ University of Bordeaux, CNRS, IMN, UMR Bordeaux France; ^6^ Clinical Investigation Center CIC1436, Methodology, Data‐Management and Statistical Analysis Unit; Department of Clinical Pharmacology, University of Toulouse, CHU of Toulouse Toulouse France; ^7^ Département Biophysique et médecine nucleaire Université de Toulouse Toulouse France; ^8^ Clinical Investigation Center, Centre Hospitalier Universitaire de Toulouse Toulouse France; ^9^ Service de Neurologie, Département de Neurologie, Hôpitaux Universitaires de Strasbourg, Hôpital de Hautepierre Strasbourg France; ^10^ Service de Neurologie, CHU de Clermont‐Ferrand Clermont‐Ferrand France; ^11^ Department of Neurology, NS‐Park/FCRIN Network Université Aix Marseille, AP‐HM Marseille France; ^12^ Department of Neurology Lille University, INSERM UMRS_1172, University Hospital Center, LICEND COEN Center Lille France; ^13^ Sorbonne Univeristé, Institut du Cerveau‐Paris Brain Institute, Assistance Publique Hôpitaux de Paris, INSERM, CNRS, Pitié‐Salpêtrière Hospital Department of Neurology, Centre d'Investigation Clinique Neurosciences, Sorbonne Université Paris France; ^14^ Lyon University Hospital, Pierre Wertheimer Neurological Hospital Neurology Department C, Expert Center for Parkinson's Disease, NS‐PARK/FCRIN Network Bron France; ^15^ Claude Bernard University Lyon 1 CNRS, INSERM, Lyon Neuroscience Research Center CRNL U1028 UMR5292, PATHPARK Bron France; ^16^ Lyon Sud Charles Mérieux Faculty of Medicine and Midwifery, Claude Bernard University Lyon 1 Oullins France; ^17^ Hospices Civils de Lyon, Hôpital Louis Pradel, Service de Médecine Nucléaire Bron France; ^18^ University Hospital of Nantes, INSERM, CIC1413 Nantes France; ^19^ Service de Neurologie, Hôpital Central, CHRU Nancy France; ^20^ King's College London UK; ^21^ Ono Pharma UK Ltd London UK

## Abstract

**Objective:**

The objective of this study was to characterize changes in candidate biomarkers in early multiple system atrophy (MSA) and identify baseline predictors of faster progression.

**Methods:**

This 1‐year, multicenter, prospective study assessed clinical, neuroimaging (3T‐magnetic resonance imaging [MRI], dopamine transporter single‐photon emission computed tomography [DaT‐SPECT]), and neurofilament light chain (NfL) changes in patients with early MSA (< 5 years from symptom onset) and healthy controls (HCs). Clinical and biomarker changes from baseline to 6 months (M6) and 12 months (M12) were analyzed. Survival status was collected at 24 months. Mixed linear regression analyzed repeated measures, whereas univariate regression identified biomarkers linked to progression. Sample size simulations were conducted for future trials.

**Results:**

Forty‐one patients with MSA and 20 HCs were included in this study. The Unified Multiple System Atrophy Rating Scale (UMSARS)‐I + II scores worsened (mean percent change from baseline was 19.8% at M6; 95% confidence interval [CI] = 13.3 to 26.4% and 31.1% 95% CI = 24.9 to 37.2% at M12). Patients with MSA showed increased cerebellar white matter and pons atrophy (M6 = −5.9 to −2.8% and M12 = −9 to −4.9%) and decreased striatal specific binding ratio (SBR; M6 = −15.8 to −7.9% and M12 = −24 to −10.4%). Patients with multiple system atrophy parkinsonian (MSA‐P) exhibited greater striatal SBR reduction, whereas patients with multiple system atrophy cerebellar (MSA‐C) had greater cerebellar and pons atrophy, evident at M6. Baseline brainstem and pons volume predicted clinical worsening at M6, whereas SBR predicted worsening at M12. Higher plasma NfL levels correlated with early dropout (14% at M12), worse UMSARS scores, lower SBR, and increased mortality risk within 24 months.

**Interpretation:**

Neuroimaging changes occur within 6 months in early MSA. High plasma NfL levels are linked to increased mortality and dropout risk. Longitudinal biomarker assessments provide valuable insights into disease progression. ANN NEUROL 2026;99:96–113

Multiple system atrophy (MSA) is a rare, sporadic, progressive, adult‐onset neurodegenerative disorder characterized by a combination of autonomic failure, parkinsonism, cerebellar ataxia, and pyramidal signs.^1^ MSA disease progression is fast, with median survival ranging between 6 and 10 years after symptom onset, even if slower progression and longer survival for more than 15 years have been reported in a minority of patients.^1^, ^2^ Whereas some symptomatic treatments exist, neuroprotective treatments for MSA remain an urgent, unmet need.^3^

The success of clinical trials for therapies with potential disease‐modifying efficacy is greatly facilitated by the availability of sensitive and reliable markers of disease progression. At present, there are no established objective and standardized biomarkers to monitor the progression of MSA. Clinical trials in MSA typically rely on clinical rating scales, such as the Unified MSA Rating Scale (UMSARS)^4^ as primary endpoint, since, ultimately, the goal of a therapeutic intervention is to preserve or improve the patients’ everyday functioning. However, UMSARS score progression rates are highly variable, as exemplified in natural history studies^2^, ^5^ and clinical trials.^6^, ^7^, ^8^, ^9^

There is a wealth of literature supporting selected candidate biomarkers of MSA, including both neuroimaging^10^, ^11^, ^12^, ^13^, ^14^, ^15^, ^16^ and wet biomarkers.^17^, ^18^, ^19^ However, most of the efforts have focused on diagnostic markers to differentiate MSA from other parkinsonian syndromes, whereas prospective longitudinal investigations, which are crucial to assess putative disease‐modifying therapies, remain sparse. Recent evidence suggests that changes in brain volume can be detected in MSA within 6 months,^11^ suggesting that such a short timeframe might be sufficient to detect indices of treatment effects.

This study was designed to characterize in a systematic manner in patients with MSA the changes after 6 and 12 months of selected candidate biomarkers derived from magnetic resonance imaging (MRI), dopamine transporter single‐photon emission computed tomography (DaT‐SPECT), blood, and cerebrospinal fluid (CSF) neurofilament light chain (NfL) measurements. Simultaneously, we assessed in the same patients the changes over time in various clinical scales commonly used to assess the symptoms and health‐related quality of life (HR‐QOL) in MSA. We also aimed at identifying baseline biomarker profiles predictive of faster clinical progression, revealing new insights for patient stratification strategies and reduction of heterogeneity. Analysis was adjusted for clinical phenotypes, that is, MSA cerebellar (MSA‐C) and MSA parkinsonian (MSA‐P), to disclose different patterns of disease progression. Implications of the results for the design of future trials in terms of sample size, trial duration, and patient sub‐types are also explored and discussed.

## Material and Methods

### Study Design

This was a non‐interventional, case–control, prospective, longitudinal, 1 year, multicenter study to describe the natural history of MSA on a panel of clinical endpoints, neuroimaging, and selected biomarkers.

### Subjects

From May 2020 to September 2021, patients diagnosed with possible or probable MSA‐P or MSA‐C^20^ were enrolled in the study through the French MSA National Reference Centre for Rare Diseases of Toulouse and Bordeaux and the associated competence centers of Paris, Lille, Strasbourg, Marseille, Clermont‐Ferrand, Lyon, Nantes, and Nancy, all belonging to the NS‐PARK/F‐CRIN network. Inclusion criteria targeted individuals aged 30 to 80 years, at an early stage of the disease, that is, less than 5 years from the onset of the first symptom (of parkinsonism, ataxia, or dysautonomia; orthostatic hypotension [OH] or urinary dysfunction), with an anticipated survival of at least 3 years based on the investigators’ judgment and with no major speech, gait, and fall disabilities (score of ≤ 2 on UMSARS items 1, 7, and 8, respectively) which could have compromised patients’ assessment and study retention.

The healthy controls (HCs) with no neurological disease matched for sex and age were recruited in the same centers with a ratio of 2:1. The HCs were included to account for physiological effects of aging on the neuroimaging biomarkers.

Participants were excluded if they presented significant cognitive impairment (Montreal Cognitive Assessment [MoCA] score < 21) or contraindications to MRI/SPECT imaging procedures.

The study was registered on ClinicalTrials.gov (NCT04229173). All participants signed written informed consent after ethics approval was obtained by the Comité de Protection des Personnes (CPP) of the Sud Mediterranée III. The study was led and sponsored by the Toulouse University Hospital.

### Clinical Assessments

Patients with MSA underwent the following clinical assessment at baseline (M0), 6 months (M6), and 12 months (M12) follow‐up: UMSARS I, II, III, and IV,^4^ COMPASS‐31,^21^ the MSA–Quality of Life questionnaire (MSA‐QOL),^22^, ^23^ MoCA version 7.1,^24^ and the Beck Depression Inventory (BDI).^25^ MoCA and BDI were administered also to HCs at M0, M6, and M12. Survival status, that is, the date of death or the date of the last visit have been also collected on September 2024 (24 months after study ending). Changes in symptomatic treatment were permitted, consistent with the observational study design.

### MRI and DaT SPECT: Procedure and Analysis

All participants underwent a 3T MRI at M0, M6, and M12, including T1‐3D, diffusion tensor imaging (DTI) sequences. MRI sequence harmonization was coordinated with the support of the French NS‐Park network by the Center for Automated Treatment of Images (CATI).^26^ Toulouse Neuroimaging Center (https://tonic.inserm.fr/) centralized and analyzed the MRI data.

DaT‐SPECT was performed in patients with MSA at M0, M6, and M12, and in HCs at M0 and M12.

See Supplementary Information for details on MRI and DaT‐SPECT data analysis.

### Neurofilament Light Chain Measurements

Plasma and CSF (lumbar puncture was proposed as an optional assessment) NfL concentrations were measured in duplicates as part of the Neuro‐4‐plexA (Simoa) kit (NF‐Light, Quanterix) on a Simoa HD‐X Analyzer (Quanterix) according to the manufacturer's instructions. Samples were diluted prior to measurement (plasma = 1:4 and CSF = 1:40). The concentrations were within the functional upper (plasma = 140 pg/ml and CSF = 14,080 pg/ml) and lower (plasma = 1.544 pg/ml and CSF = 15.44 pg/ml) limits of quantification except for one CSF sample we were unable to quantify. All blood and CSF samples from collaborating centers were tested at the Plateforme Analytique de Recherche en Santé (PARS; Bordeaux University Hospital), using one batch of reagents (lot 503770). All NfL values were within the linear range of the assay. For plasma, the mean intra‐assay coefficient of variation (CV) of duplicate determinations for concentration was 4.5%. In the CSF, the mean intra‐assay CV was 3.8%. Of note, an extensive panel of blood and CSF biomarkers other than NfL has been also assessed, including α‐syn, YKL‐40, p181‐tau, total‐tau, and GFAP. Those data will be presented in a separate paper.

### Statistical Analysis

Descriptive analyses were performed on the demographics, clinical and concomitant medications for parkinsonism, and OH data collected at baseline, and on all clinical and biomarker data collected across all 3 timepoints (baseline, M6, and M12) in both the HCs and the patients with MSA patients, considered as a group (MSA‐P + C) and stratified by MSA‐P and MSA‐C subtypes. Mean values with standard deviation (SD) were calculated for each group, and standardized differences for mean and proportion were calculated between the HCs and the patients with MSA‐P + C, and between patients with MSA‐P and MSA‐C. Change over time in patients and HCs were calculated in terms of absolute change and percent change from baseline to M6 (M0–M6), from baseline to M12 (M0–M12), and from M6 to M12 (M6–M12). The 95% confidence intervals (CIs) were calculated for the mean of the percent change for clinical and biomarker data in both HCs and patients with MSA (as a whole group and stratified by patients with MSA‐P and MSA‐C). Differences in percent changes between groups and subtypes were evaluated using the Mann–Whitney‐Wilcoxon Test. Mixed linear regression models for repeated measures were performed with crude values as response variables and group (MSA‐P / MSA‐C), visit (M0, M6, and M12), and their interaction as explanatory variables. Models were adjusted for age and sex. Mixed models for repeated measures were used, with the response variable corresponding to the parameter of interest. Explanatory variables included group, visit, and the interaction between group and visit. All models were adjusted for age and sex. A compound symmetry covariance structure was applied, and models were estimated using the Restricted Maximum Likelihood (REML) method. Model convergence and the normality of residuals were assessed.

Cross‐sectional and longitudinal correlations between total UMSARS‐I + II/MSA‐QOL and biomarkers were estimated using the Spearman coefficient with 95% CIs.

To identify baseline biomarkers associated with clinical progression, univariate linear regression models were performed using the crude delta of the UMSARS‐I + II at M6 (M0–M6) and M12 (M0–M12) as the response variable and selected biomarkers as explanatory variables. Each univariate model was further adjusted for MSA subtype and disease duration. Due to the limited number of patients, no multivariable analyses were performed.

To further characterize specific patient profiles, differences in baseline clinical and biomarker values between the following patient sub‐groups were examined with the Mann–Whitney‐Wilcoxon Test: (1) slow versus fast progressors, defined by a cutoff score based on the median delta of the total UMSARS‐I + II between M0 and M12 among all patients with MSA, (2) patients having completed the study versus premature dropouts (before M12), and (3) patients with high versus low NfL values, considering the baseline median as threshold. Survival analyses tested the association between NfL levels (based on median baseline values) and death within the 24 months after study enrollment. Differences in the estimated survival distribution were examined using the log rank test.

To inform future clinical trial designs, sample size simulations were performed with R software version 3.5.1. Standardized effect sizes were calculated by dividing the annual mean change by the SD of the annual mean change for each of the parameters studied. Sample size calculations were based on a 2‐sided significance level (alpha) of 5%, and a power (1‐beta) of 80%, assuming hypothetical 30% and 50% reductions in the progression of each parameter, respectively. Only parameters showing longitudinal changes were selected for sample size calculation.

To preserve the integrity of the original data and the transparency of an observational study, missing data were not imputed.

The level of significance was set at *p* < 0.05. Statistical analyses were conducted using SAS version 9.4 software. Based on the observational descriptive nature of this pilot study, which prioritized discovery over strict precision, no adjustments for multiple comparisons were performed. Significant findings are intended to guide future confirmatory studies, better powered, and with appropriate adjustments, in the formulation of their objectives and study design.

## Results

Baseline demographic and clinical features are detailed in Tables 1 and 2.

**TABLE 1:** Demographic and Clinical Data of Healthy Controls and Patients With MSA at Baseline

	HCs	MSA‐P + C	MSA‐P	MSA‐C	MSA‐P vs MSA‐C	HC vs MSA‐(P + C)
Characteristics	(N = 20)	(N = 41)	(N = 26)	(N = 15)	SD	SD
Age, yr	63.7 (5.8)	63.0 (7.5)	63.8 (7.7)	61.5 (7.3)	**0.27**	−0.09
Sex, F	11 (55%)	21 (51.2%)	15 (57.7%)	6 (40%)	**−0.36**	0.08
BMI, Kg/m^2^	27.5 (4.3)	25.9 (4.9)	25.4 (5.4)	26.6 (4.0)	**−0.31**	**−0.44**
Disease duration, mo	NA	41.8 (14.9)	41.7 (14.2)	41.8 (16.6)	0.14	NA
Diagnostic certainty at baseline, n (%)	NA					NA
Probable		34 (82.9)	20 (76.9)	14 (93.3)		
Possible		7 (17.1)	6 (23.1)	1 (6.7)		
UMSARS	NA					NA
I		17.7 (4.3)	18.7 (4.4)	15.9 (3.5)	**0.67**	
II		19.1 (6.1)	20.1 (5.1)	17.5 (5.1)	**0.39**	
I + II		36.8 (9.4)	38.8 (10.1)	33.3 (7.3)	**0.61**	
III						
Delta systolic BP^a^		17.4 (19.2)	18.2 (21.8)	15.9 (14.4)	**0.2**	
Delta diastolic BP^a^		6.1 (15.5)	6.7 (16.7)	5.1 (13.8)	0.07	
IV, n (%)						
1		8 (19.5)	6 (23.1)	2 (13.3)	**0.25**	
2		22 (53.7)	13 (50.0)	9 (60.0)	**−0.2**	
3		8 (19.5)	5 (19.2%)	3 (20.0)	−0.02	
4		3 (7.3)	2 (7.7)	1 (7.7)	0.04	

Values are presented as mean (SD), if not otherwise specified. (*): delta of systolic and diastolic BP between lying and after 3 minutes of standing. The SS = the difference in means or proportions divided by standard error; the figures in bold correspond to the imbalance defined as absolute value greater than 0.20.

BMI = body mass index; BP = blood pressure; HCs = healthy controls; MSA = multiple system atrophy; MSA‐C = multiple system atrophy cerebellar; MSA‐P = multiple system atrophy parkinsonian; MSA‐P + C = multiple system atrophy parkinsonian and cerebellar; NA = not applicable; SD = standard deviation; UMSARS = Unified Multiple System Atrophy Rating Scale.

**TABLE 2:** Clinical and Therapeutic Data of Healthy Controls and Patients With MSA at Baseline

	HCs	MSA‐P + C	MSA‐P	MSA‐C	MSA‐P vs MSA‐C	HC vs MSA‐P + C
Characteristics	(N = 20)	(N = 41)	(N = 26)	(N = 15)	SD	SD
MSA‐QOL	NA					
Total score		38.9 (15.6)	41.2 (17.4)	33.6 (10.7)	**0.5**	
Motor sub‐score		41.5 (16.7)	43.9 (18.4)	37.5 (12.9)	**0.38**	
Non‐motor sub‐score		37.8 (15.1)	42.0 (14.4)	30.4 (13.9)	**0.74**	
Emotional sub‐score		36.3 (22.9)	38.6 (25.2)	32.4 (18.4)	0.19	
MoCA	27.9 (1.6)	27.1 (2.4)	27.1 (2.5)	26.9 (2.4)	0.09	**−0.33**
BMI	5.3 (7.0)	15.2 (7.7)	15.3 (7.8)	15.1 (7.8)	−0.01	**1.47**
Compass‐31	NA					NA
Total score		35.4 (16.8)	35.9 (16.3)	34.5 (18.2)	0.1	
Bladder sub‐score		4.2 (2.7)	4.3 (2.8)	3.9 (2.4)	0.15	
Gastrointestinal sub‐score		6.7 (3.3)	7.7 (2.7)	4.9 (3.7)	**0.79**	
Orthostatic intolerance sub‐score		17.2 (13.4)	16.0 (12.5)	19.2 (15.1)	**−0.29**	
Pupillomotor sub‐score		1.9 (1.1)	2.0 (1.2)	1.8 (1.0)	**0.2**	
Secretomotor sub‐score		5.1 (3.6)	5.4 (3.8)	4.6 (3.3)	**0.25**	
Vasomotor sub‐score		0.3 (1.0)	0.4 (1.1)	0.2 (0.7)	**0.28**	
Dopaminergic therapy, n (%)	0 (0.0)	25 (61.0)	22 (84.6)	3 (20.0)	**1.70**	**1.77**
Treatment for orthostatic hypotension, n (%)	0 (0.0)	9 (22.0)	6 (23.1)	3 (20.0)	0.07	**0.75**
Plasma NfL chain, pg/ml	NA	39.3 (20.8)	44.3 (23.9)	30.6 (9.5)	**0.68**	NA

Standardized difference = difference in means or proportions divided by standard error; the figures in bold corresponds to the imbalance defined as absolute value greater than 0.20.

BMI = body mass index; HCs = healthy controls; MoCA = Montreal Cognitive Assessment; MSA = multiple system atrophy; MSA‐C = multiple system atrophy cerebellar; MSA‐P = multiple system atrophy parkinsonian; MSA‐P + C = multiple system atrophy parkinsonian and cerebellar; MSA‐QOL = Multiple System Atrophy Quality of Life questionnaire; NA = not applicable; NfL = neurofilament light chain; SD = standard deviation.

Forty‐one patients with MSA (21 were female patients) were enrolled in the study. Mean (SD) age and disease duration (time elapsed since the onset of the first symptom, of parkinsonism, ataxia, or dysautonomia) were 63.0 ± 7.5 years and 41.8 ± 14.9 months, respectively. At inclusion, 34 patients (82.9%) had probable MSA and 26 patients (63.4%) had MSA‐P. Twenty HCs matched for sex and age were also included.

**FIGURE 1:**
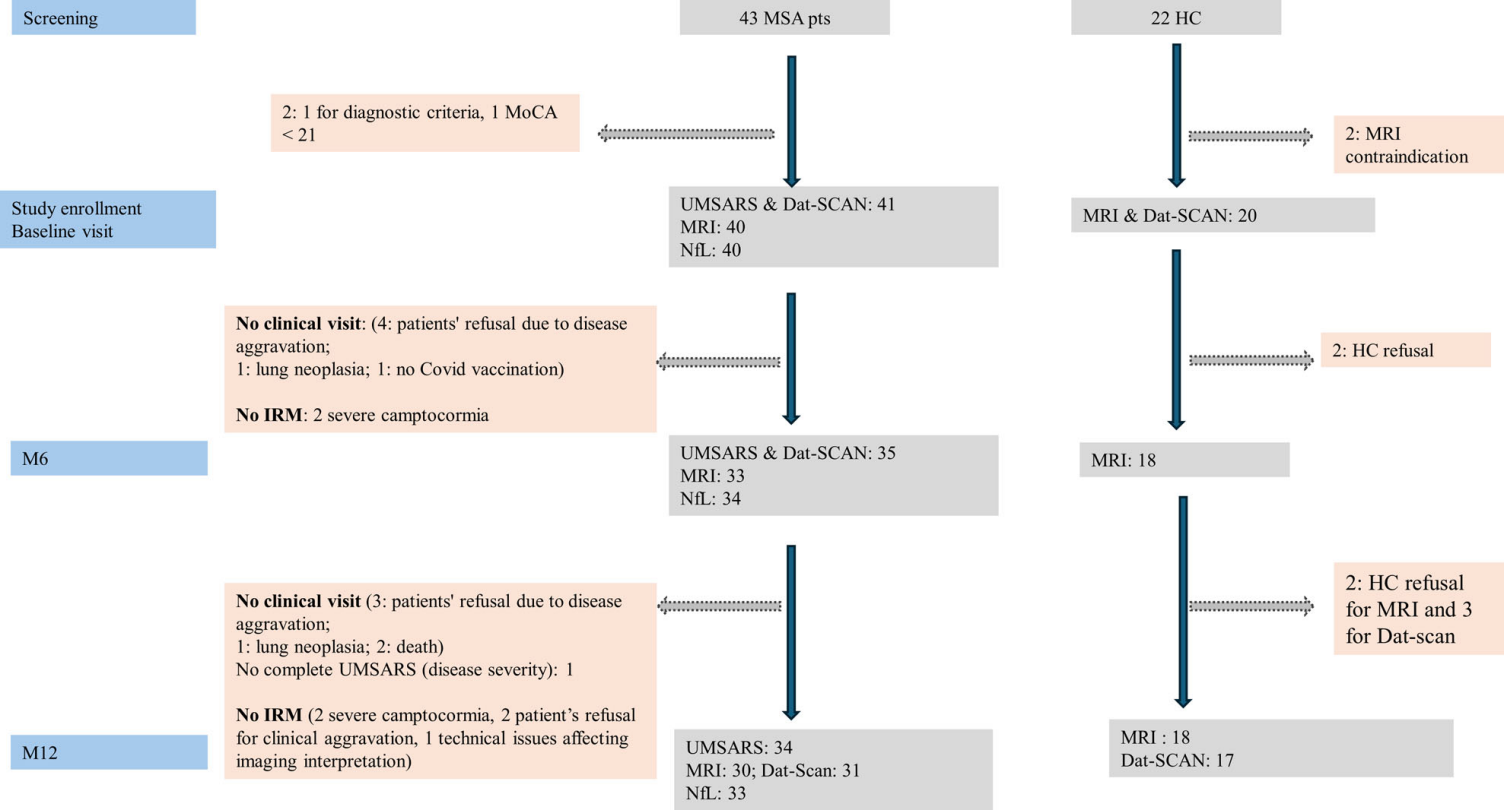
Study flowchart. [Color figure can be viewed at www.annalsofneurology.org]

Clinical and neuroimaging data were collected at baseline in all 41 patients. At M6 and M12, clinical data were collected in 35 patients with MSA‐P + C (2 patients died between M6 and M12, 4 withdrew from the study; at M12, one patient performed the clinical assessment but only partially completed the UMSARS), whereas MRI and DaT‐SPECT data were collected in 33 and 29 patients at M6 and M12, respectively (worsening of clinical disability was the cause to not perform clinical or neuroimaging assessments in about three quarters of the patients; see the Flowchart in Fig 1). Blood/CSF NfL was collected for 34/8, 34/6, and 33/5 patients with MSA‐P + C at baseline, M6, and M12, respectively. Considering the low sample size for CSF NfL and its known correlation with plasma values,^17^ only the latter values are reported here.

### Clinical Data and Biomarkers at Baseline and Over Time

#### Clinical Parameters

**FIGURE 2:**
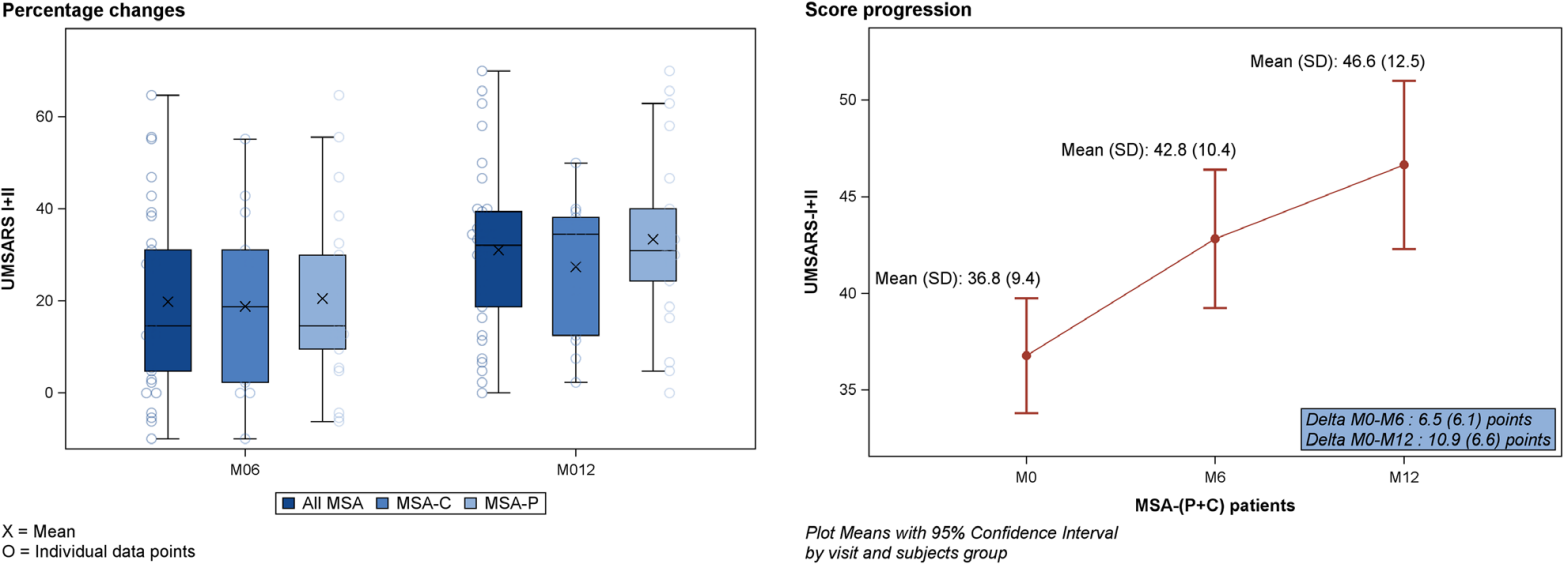
Longitudinal percentage change and crude delta progression for total UMSARS I + II among all patients with MSA‐P + C. MSA‐P + C = multiple system atrophy parkinsonian and cerebellar; UMSARS = Unified Multiple System Atrophy Rating Scale. [Color figure can be viewed at www.annalsofneurology.org]

Longitudinal worsening was observed in total UMSARS‐I + II scores, with a mean (SD) increase in the total score of +6.5 (6.1) and +10.9 (6.6) points at M6 and M12, respectively (Fig 2, Tables 3 and 4, and Supplementary Table S1). No significant longitudinal changes were noted over 1 year in UMSARS‐III. A slight increase in UMSARS‐IV was observed when considering absolute values, with a mean increase of nearly +49% at 12 months when considering percent changes from baseline.

**TABLE 3:** Biomarkers Progression (Percentage Changes) for Patients With MSA‐P + C Versus Healthy Controls

							MSA‐P + C vs HCs		
	HCs			MSA‐P + C			M0–M6	M0–M12	M6–M12
Characteristics	M0–M6	M0–M12	M6–M12	M0–M6	M0–M12	M6–M12	MWW	MWW	MWW
Clinical									
UMSARS									
I	NA	NA	NA	22.7 (22.7)	34.8 (21.6)	14.7 (17.8)	NA	NA	NA
II	NA	NA	NA	18.5 (20.7)	29.2 (24.6)	10.9 (22.2)	NA	NA	NA
I + II	NA	NA	NA	19.8 (19)	31.1 (17.7)	12.3 (15.2)	NA	NA	NA
IV	NA	NA	NA	27.1 (62.8)	49.8 (61.5)	24.2 (33.2)	NA	NA	NA
MSA‐QOL, total	NA	NA	NA	21.5 (42.2)	31.6 (36)	13.1 (32)	NA	NA	NA
Motor	NA	NA	NA	30.3 (48.2)	51.6 (55.1)	22.1 (45.2)	NA	NA	NA
Non‐motor	NA	NA	NA	21.3 (45.8)	23.4 (32.5)	14.9 (52.1)	NA	NA	NA
Emotional	NA	NA	NA	30.6 (85.1)	36.1 (89.5)	8.9 (52.3)	NA	NA	NA
MRI ‐ regional atrophy									
Putamen	−0.6 (1.9)	−0.6 (1.6)	0 (1.1)	−1.5 (2.5)	−3.3 (3.9)	−1.7 (2.9)	0.2464	**0.0087**	**0.0218**
Caudate nucleus	−0.5 (1.6)	−0.6 (1)	−0.1 (1.3)	−1.9 (2.8)	−2.9 (4.7)	−1.1 (3.5)	**0.0427**	**0.0294**	0.4412
Total white matter	−0.1 (1)	−0.5 (1)	−0.4 (1)	−0.6 (1.4)	−1.1 (1.3)	−0.7 (1.2)	0.2967	0.1012	0.2477
Total gray matter	0 (1.7)	−0.4 (1.2)	−0.3 (1.2)	−1 (1.8)	−1.6 (1.6)	−0.7 (1.1)	**0.0168**	**0.0056**	0.4941
Mean striatum	−0.5 (1.7)	−0.6 (1.2)	0 (0.9)	−1.7 (2.2)	−3.2 (3.6)	−1.5 (2.7)	0.0805	**0.0056**	0.0966
Cerebellum white matter	−0.4 (2.7)	−1 (2.1)	−0.6 (2.3)	−4.4 (4.3)	−7 (5.5)	−3.4 (4.6)	**0.0059**	**0.0003**	**0.0271**
Cerebellum gray matter	0.7 (1.8)	−0.1 (1.5)	−0.7 (0.9)	−1.6 (2.1)	−3.3 (2.4)	−1.6 (2.1)	**0.0007**	**0.0001**	**0.0422**
Medulla	0.3 (3.6)	0.1 (2.9)	−0.2 (2.9)	−0.7 (3.6)	−1.6 (2.6)	−0.5 (2.6)	0.1234	0.0548	0.9913
Pons	−0.4 (1.4)	−0.5 (1.7)	−0.1 (1.1)	−3.2 (2.3)	−5.8 (3.7)	−2.9 (2)	**0.0001**	**< 0.0001**	**< 0.0001**
Midbrain	−0.5 (1.8)	−0.9 (1.7)	−0.3 (1.5)	−1.1 (2.8)	−2 (2.4)	−1.3 (2.2)	0.3741	0.0892	0.192
Brainstem	−0.3 (1.6)	−0.5 (1.8)	−0.2 (1.2)	−2.1 (1.6)	−3.8 (2.4)	−1.9 (1.5)	**0.0011**	**0.0001**	**0.0008**

Results are expressed as mean (SD). Standardized deviation = difference in means or proportions divided by standard error; the figures in bold corresponds to the imbalance defined as absolute value greater than 0.20.

HC = healthy controls; M0 = baseline; M6 = month 6; M12 = month 12; MRI = magnetic resonance imaging; MSA‐P + C = multiple system atrophy parkinsonian and cerebellar; MSA‐QOL = Multiple System Atrophy Quality of Life questionnaire; MWW = Mann–Whitney‐Wilcoxon test; NA = not applicable; UMSARS = Unified Multiple System Atrophy Rating Scale.

**TABLE 4:** Biomarkers Progression (Percentage Changes) for Patients With MSA‐P + C Versus Healthy Controls

							MSA‐P + C vs HCs		
	HCs			MSA‐P + C			M0–M6	M0–M12	M6–M12
Characteristics	M0–M6	M0–M12	M6–M12	M0–M6	M0–M12	M6–M12	MWW	MWW	MWW
MRI ‐ mean diffusivity									
Putamen	0.2 (2.7)	0.6 (2.4)	0.7 (2.9)	2.1 (2.6)	2.2 (3.8)	0.7 (3.1)	**0.0077**	0.1197	0.739
Caudate nucleus	0.5 (3.4)	1.8 (4)	1.7 (4.2)	2.7 (4.9)	5.3 (7.9)	2.9 (7.1)	0.1725	0.1341	0.7184
Striatum	0.6 (2.1)	1.2 (2.5)	1 (2.8)	2.4 (3.5)	3.7 (5.3)	1.8 (4.5)	0.0974	0.1454	0.7916
Cerebellum white matter	0.7 (2.7)	0.6 (3.4)	−0.1 (3)	2 (3.4)	0.7 (4.7)	−0.4 (3.8)	**0.0227**	0.6867	0.8653
Cerebellum gray matter	1.2 (2.5)	1 (3.7)	−0.2 (3.5)	2.5 (3.6)	2.4 (4.3)	0.4 (3.6)	**0.0436**	0.3082	0.5617
Medulla	0.7 (5.3)	3.3 (5.2)	3.1 (4.7)	2.2 (6.9)	2.1 (7.8)	0.5 (5.8)	0.482	0.6647	0.1145
Pons	0.7 (5.8)	1.5 (4.6)	0.7 (5.6)	1.3 (5.8)	1.4 (6.5)	1.5 (5.1)	0.4291	0.7559	0.4616
Midbrain	1.1 (4.6)	1.7 (4.5)	0.7 (3.9)	0.8 (4.2)	2.4 (4.4)	1.9 (3.9)	0.8643	0.615	0.1613
Brainstem	0.4 (4.9)	2.3 (3.3)	2 (5)	1.3 (4.9)	1.2 (4.7)	0.8 (4.7)	0.322	0.482	0.8628
MRI ‐ fractional anisotropy									
Total white matter	−1.3 (3.3)	−0.8 (5.8)	0.6 (6.2)	−1.7 (2.9)	−0.6 (7.5)	1 (7)	0.6726	0.8667	0.8268
Cerebellar white matter	−1.3 (3.4)	−1 (6.1)	0.3 (5.7)	−2.3 (4.4)	−2.7 (6)	−0.5 (6)	0.2907	0.2542	0.6447
Datscan									
caudate nucleus – SBR	NA	0.8 (13.2)	NA	−11 (14.6)	−14 (22.7)	−3.5 (24)	NA	**0.0313**	NA
Putamen– SBR	NA	−2.5 (13.8)	NA	−8.8 (24.5)	−19.3 (21.3)	−11.5 (19.1)	NA	**0.0036**	NA
Striatum	NA	−1 (11.4)	NA	−11.9 (11.5)	−17.2 (18.6)	−6.9 (18.9)	NA	**0.0024**	NA
Putamen vs caudate ratio	NA	−2.6 (12.9)	NA	7.2 (43.9)	3.5 (63.7)	−2.7 (35.6)	NA	0.1326	NA
Wet biomarkers									
Plasma NfL chain	NA	NA	NA	8.9 (22.9)	19 (41.4)	9.5 (30.9)	NA	NA	NA

Results are expressed as mean (SD). Standardized deviation = difference in means or proportions divided by standard error; the figures in bold corresponds to an imbalance defined as absolute value greater than 0.20.

HC = healthy controls; M0 = baseline; M6 = month 6; M12 = month 12; MRI = magnetic resonance imaging; MSA‐P + C = multiple system atrophy parkinsonian and cerebellar; MWW = Mann–Whitney‐Wilcoxon test; NA = not applicable; NfL = neurofilament light chain; SBR = specific binding ratio.

MSA‐QOL motor, non‐motor, and emotional sub‐scores all worsened significantly from M0 to M6 (+30%, +21%, and +30%, corresponding to a delta mean of 8.3 [12.6], 5.3 [13.9], and 2.3 [18] points, respectively) and from M0 to M12 (+51%, +23%, and +36% corresponding to a mean of 14.7 [11.5], 6.2 [11.3], and 3.1 [18.1] points, respectively; Supplementary Table S2).

At baseline, patients with MSA‐P had significantly higher total UMSARS‐I + II scores, along with higher disability (UMSARS‐IV), worse dysautonomia (COMPASS‐31 scores, except for the bladder domain), and worse HR‐QOL on motor and non‐motor sub‐scores versus patients with MSA‐C (see Tables 1 and 2). MoCA and BDI scores were significantly higher in patients with MSA‐P + C than HCs (see Tables 1 and 2), but no consistent changes were observed over time in MOCA, BDI, and COMPASS‐31 scores (see Supplementary Table S1).

#### Magnetic Resonance Imaging

##### Volume

At baseline, a reduced regional brain volume was observed in patients with MSA‐P + C compared with HCs in the putamen, the cerebellum (grey and white matter), the pons, and the whole brainstem (see Supplementary Table S1).

Patients with MSA‐P + C showed volume loss across all regions of interest (ROIs) at M6 and M12 versus baseline (except for the medulla at M6; see Tables 3 and 4, and Supplementary Table S2a). Volume loss from baseline ranged from −4.4% (cerebellar gray matter) to −0.6% (total white matter) at M6; and from −7% (cerebellar gray matter) to −1% (total white matter) at M12.

**FIGURE 3:**
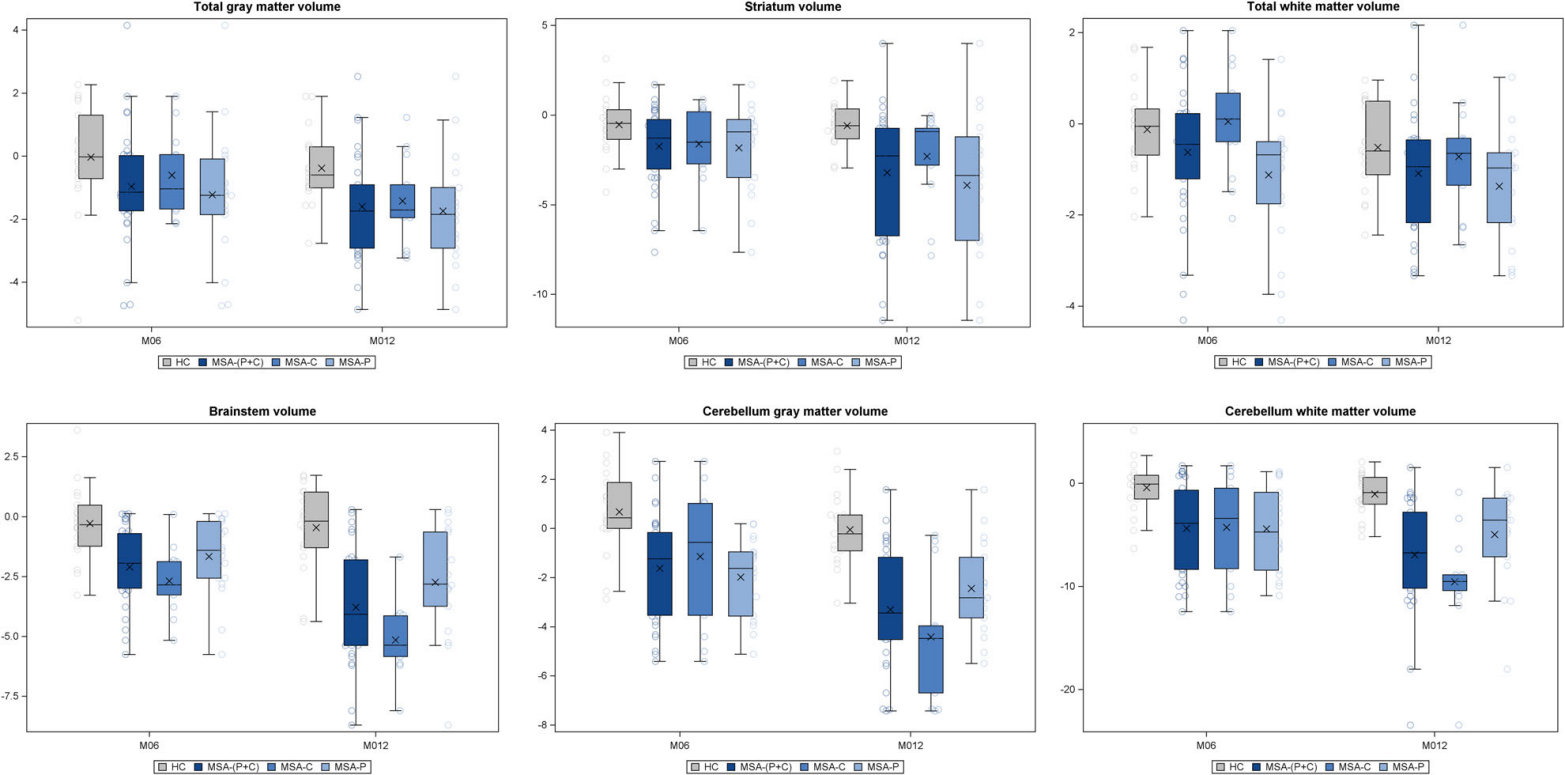
Progression of MRI atrophy at M6 and M12 (percentage changes). M6 = month 6; M12 = month 12; MRI = magnetic resonance imaging. [Color figure can be viewed at www.annalsofneurology.org]

When comparing the patients with MSA‐P + C group and the HC group, the above changes were significant at M12 for total gray matter tissue, striatum (both caudate and putamen), cerebellum, and brainstem, in particular the pons (see Tables 3 and 4, and Fig 3). Although no significant changes were observed in total white matter volume, progression of white matter atrophy was noted in the cerebellum. Changes in gray matter volume (both total and cerebellar) and cerebellar white matter volume were already significant in most regions by M6 when comparing patients with MSA‐C and patients with MSA‐P, a mixed‐model analysis revealed that atrophy was greater at M12 in patients with MSA‐C in the cerebellum gray and white matter and brainstem, more specifically in the pons (*p* = 0.0134; Supplementary Table S3). Moreover, when considering the M0 to M6 versus the M6 to M12 time periods, patients with MSA‐C showed greater progression of atrophy compared with patients with MSA‐P in the pons, brainstem, and total white matter from M0 to M6 and in the pons and cerebellum gray matter from M6 to M12. This result was further corroborated by a model adjusted for timepoints (M0, M6, and M12) and patient sub‐groups which showed a significant interaction between time and sub‐group for brain volume in the pons and cerebellum gray matter, and a trend interaction for total white matter (see Supplementary Table S3).

##### Mean Diffusivity

Compared with the HCs, patients with MSA‐P + C showed at baseline increased movement disorder mean diffusivity (MD) in cerebellum gray and white matter (see Supplementary Table S1). Relative to the HCs, patients with MSA‐P + C showed further increase in MD at M6 but not M12 in the putamen and the cerebellum (gray and white matter). No differences were observed between patients with MSA‐C and patients with MSA‐P regarding longitudinal MD changes (see Supplementary Tables S2b and S3).

##### Fractional Anisotropy

Fractional anisotropy (FA) in total white matter and cerebellar white matter was not different in patients with MSA‐P + C compared with the HCs and did not show longitudinal changes. The comparison between patients with MSA‐P and patients with MSA‐C revealed a greater reduction in FA at M12 relative to M0 in patients with MSA‐P (see Supplementary Tables S2b and S3).

##### Dopamine Transporter Single‐Photon Emission Computed Tomography Scan

**FIGURE 4:**
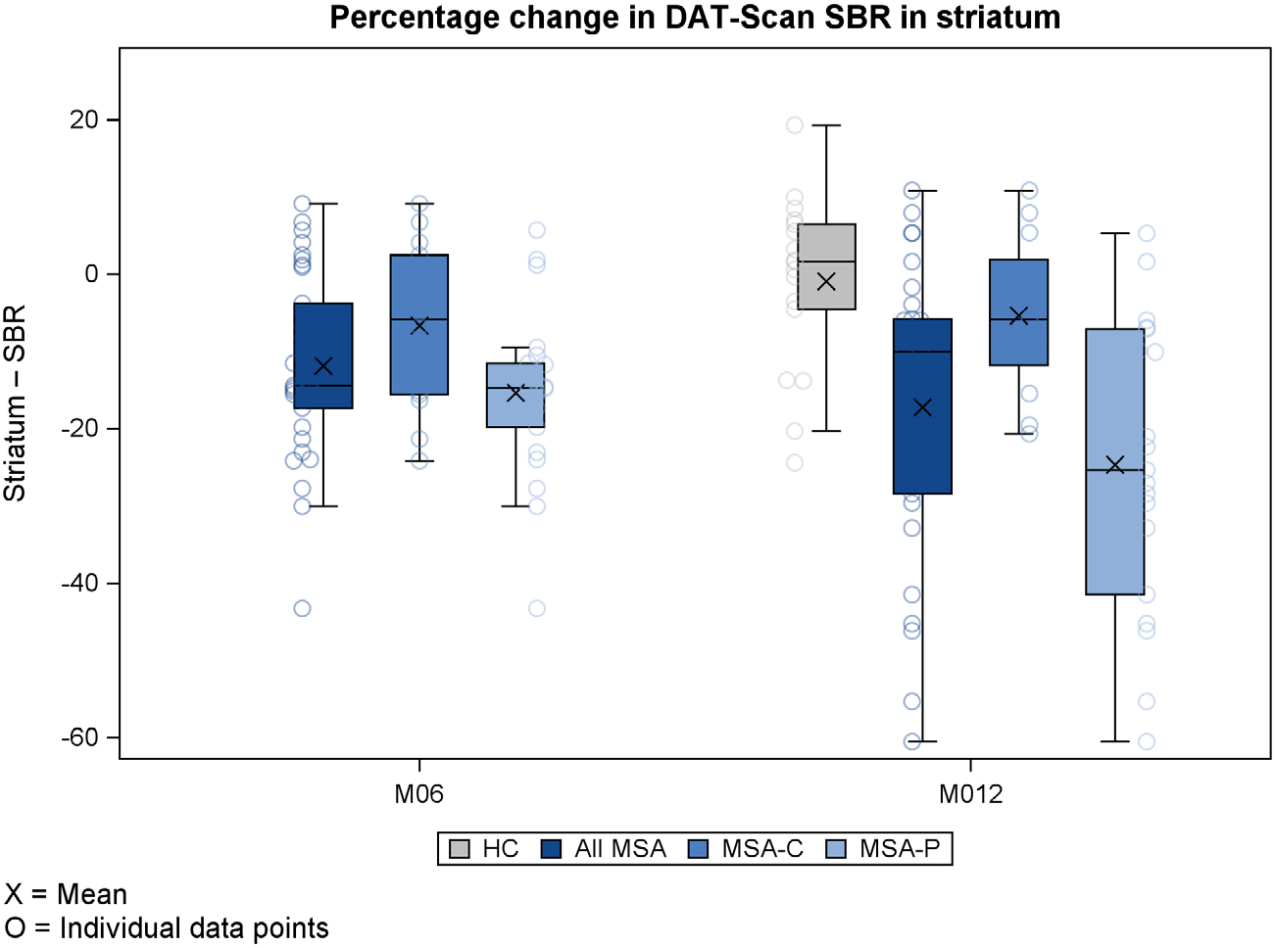
Percentage change in DaTscan SBR in striatum. SBR = specific binding ratio. [Color figure can be viewed at www.annalsofneurology.org]

Compared with the HCs, patients with MSA‐P + C had a considerably lower specific binding ratio (SBR) in the striatum at baseline (see Supplementary Table S1) and showed a decline in striatal SBR at M12 (Fig 4, see Supplementary Tables 3 and 4; of the HCs did not undergo a DaT‐SPECT at M6). Among patients with MSA‐P + C, a significant decline from baseline in SBR was observed across all striatal regions (caudate, putamen, and striatum) at M6 and M12 (see Supplementary Table S2a). This decline was significantly more pronounced in patients with MSA‐P compared with patients with MSA‐C at 12 months (see Supplementary Table S3). Putamen versus caudate ratio was stable over time. Of note also, 5 patients with MSA‐C exhibited normal DaT‐SPECT results, which remained unchanged over the duration of the study (see Supplementary Table S7 for clinical and biomarkers details of those 5 patients).

##### Plasma Neurofilament

**FIGURE 5:**
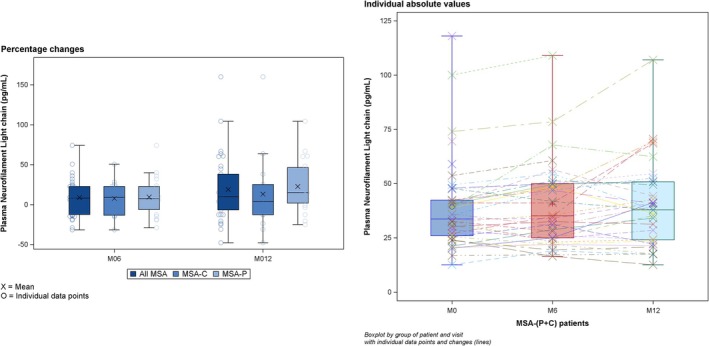
Plasma NfL progression. NfL = neurofilament light chain. [Color figure can be viewed at www.annalsofneurology.org]

Mean (SD) plasma NfL values in patients with MSA‐P + C were 39.3 (20.8) pg/ml, 40.6 (19.4) pg/ml, and 40.3 (19.9) pg/ml at baseline, M6, and M12, respectively (Fig 5). Compared with patients with MSA‐C, the patients with MSA‐P had higher plasma NfL levels at baseline (see Tables 1 and 2). The CSF NfL at M0, M6, and M12 are reported in the Supplementary Information (Supplementary Table S4).

### Correlations Between UMSARS/MSA QOL Scores and Biomarkers

Cross‐sectional correlations between total UMSARS‐I + II scores with neuroimaging biomarkers and NfL values are detailed in the Supplementary Information (Supplementary Figs S2–S4 for all timepoints and Supplementary Table S5 for M0). At baseline, M6, and M12, significant cross‐sectional correlations were found in patients with MSA‐P + C between clinical assessment of disease severity (total UMSARS‐I + II) and plasma NfL values, striatum volume, and SBR. Similar correlations were also observed at baseline in MSA‐P (but not MSA‐C; data not shown). Mild longitudinal correlations are found for the cerebellar white matter atrophy/diffusivity at M6 (*r* ^2^ = 0.384 and 0.417, respectively) and mid brain atrophy at M12 (*r* ^2^ = 0.397). Regarding QOL data, cross‐sectional significant correlations are found for putamen/striatum atrophy and DAt‐SPECT changes and NfL plasma levels (*r* ^2^ = approximately 0.55), whereas only mild longitudinal correlations are found for midbrain/brainstem atrophy and DaT‐SPECT changes at M6 (*r* ^2^ = approximately 0.33) Supplementary Figs S4 and S5.

### Baseline Predictors of Clinical Progression and Survival

At univariate analysis, baseline brainstem and pons atrophy were associated to the clinical aggravation at M6 (total UMSARS‐I + II delta score), adjusting for MSA phenotype and disease duration (95% CI = −0.001 to −0.0001; *p* = 0.019 and 95% CI = −0.009 to −0.0003; *p* = 0.01, respectively). Striatum SBR was associated to clinical worsening (total UMSARS‐I + II delta score) at M12 in unadjusted analysis (95% CI = 4.549 to −0.648; *p* = 0.010) but only a trend was observed when adjusting for MSA phenotype and disease duration (95% CI = −5.027 to −0.137; *p* = 0.0626). Likewise, a trend was pointed out for baseline NfL values in unadjusted analysis (95% CI = −0.008 to 0.365; *p* = 0.060) being associated to clinical aggravation at M12 (but adjusted values were not significant: 95% CI = −0.043 to 0.341; *p* = 0.12).

A comparison between groups stratified by the median baseline NfL value (33.7 pg/ml) revealed that patients with higher NfL values had higher total UMSARS‐I + II scores (*p* = 0.0004), worse MSA‐QOL scores (*p* = 0.0018), a more severe putamen and striatum atrophy (*p* = 0.019 and 0.013), and a more severe dopaminergic denervation as per striatal SBR (*p* = 0.009).

**FIGURE 6:**
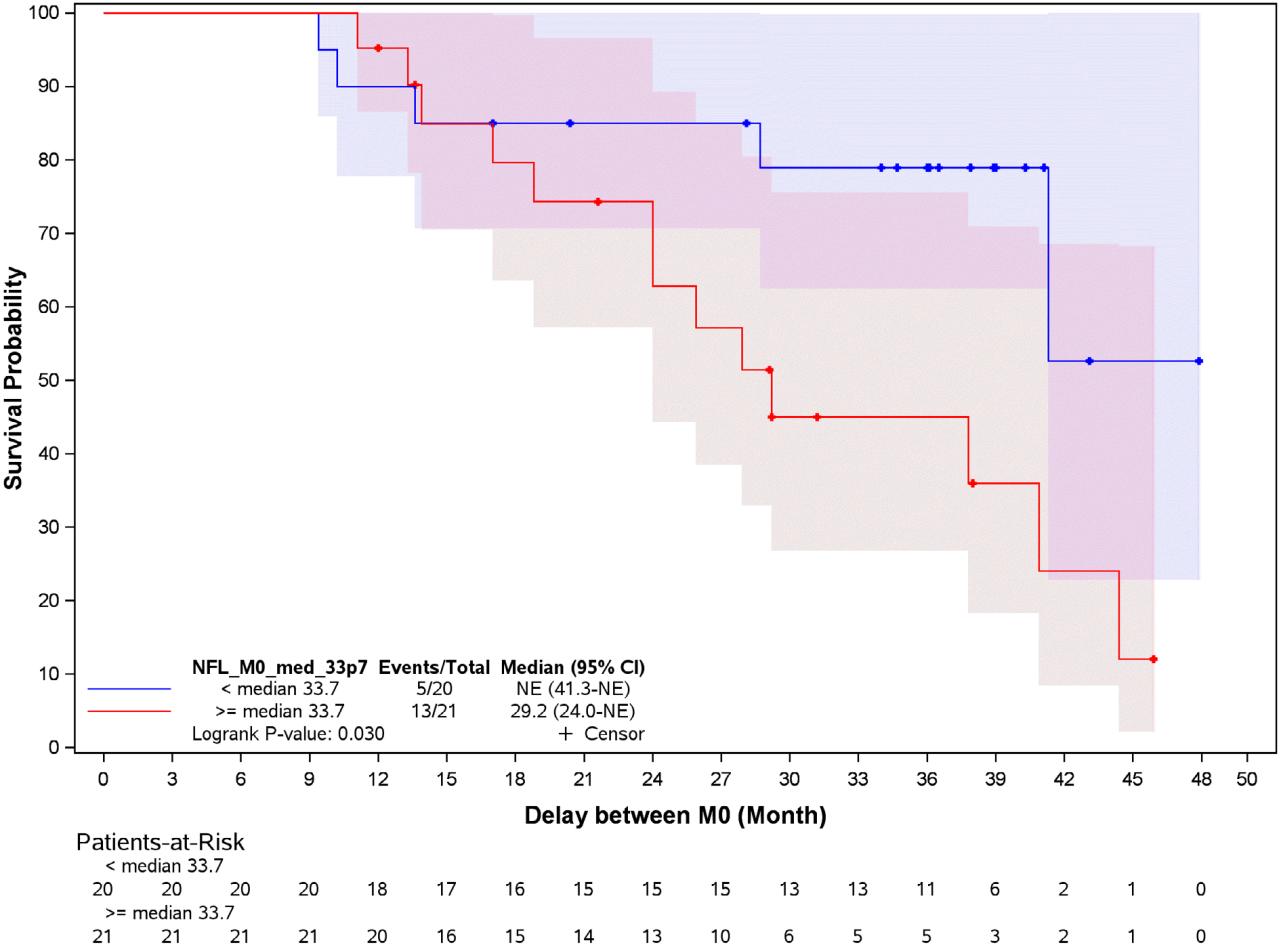
Survival curves based on baseline NfL values. NfL = neurofilament light chain. [Color figure can be viewed at www.annalsofneurology.org]

Higher plasma baseline NfL values (> 33.7 pg/ml) were associated to a shorter overall survival after 18 months (log rank = 0.003; Fig 6). No MSA phenotype was associated with shorter survival; however, the death rate among patients with MSA‐P was twice as high as that of patients with MSA‐C (see Supplementary Fig S1; log rank = 0.136).

### Comparison of Sub‐Groups Stratified by Clinical Progression (Slow Vs Fast), and Dropouts (Protocol Completion Vs Drop‐Out) on Biomarkers

A median change of 10 points on the total UMSARS‐I + II score was found between M0 and M12 and it was adopted as the cutoff to define slow and fast progressors in the study population. No baseline clinical and neuroimaging differences were found comparing slow versus fast progressors with the exception of a higher BDI score in fast progressors (*p* = 0.03; Supplementary Table S6). Patients who dropped out from the study before M12 had worse baseline MSA‐QOL, mainly due to motor symptoms and more severe depression score at BDI (*p* = 0.027 and *p* = 0.016, respectively). No differences were found for MSA phenotype (MSA‐C vs MSA‐P) comparing patients who dropped out versus the ones who completed the study or comparing slow versus fast progressors. Additionally, they had a significantly higher baseline plasma NfL value (*p* = 0.0125). Consequently, clinical and biomarkers of patients with higher plasma NfL at baseline could not be assessed at M12 (Fig 6).

### Sample Size Estimation for Clinical Trials

Table 5 displays the number of patients per group needed to detect a treatment effect of 30% or 50% reduction in progression, in a clinical trial with a follow‐up of 6 or 12 months. With the 6‐month follow‐up, the outcomes requiring the smallest sample size were the pons and brainstem volume, reducing the number of patients needed by 35% to 44% versus the total UMSARS‐I + II score, whereas with a 12‐month follow‐up, the total UMSARS‐I + II score and MRI biomarkers performed in a similar way in terms of number of patients to be enrolled (see Table 5).

**TABLE 5:** Sample Size Calculations Based on Biomarkers Natural History Progression

Measurement	Delta M0 > M6				Delta M0 > M12			
	N^a^	Standardized Effect Size^c^	Power Calculation for Clinical Trials		N	Standardized Effect Size	Power Calculation for Clinical Trials	
	Mean (SD)^b^		Subjects Per Group		Mean (SD)		Subjects Per Group	
			If 30% Reduction,	If 50% Reduction,			If 30% Reduction,	If 50% Reduction,
			Mean^d^	Mean			Mean	Mean
			N^e^	N			N	N
Total UMSARS‐I + II	N = 35	1.1	4.6	3.3	N = 34	1.7	7.6	5.5
	6.5 (6.1)		N = 162	N = 58	10.9 (6.6)		N = 63	N = 24
Striatum – DaTscan	N = 35	−1	−0.14	−0.1	N = 31	−1	−0.2	−0.15
	−0.2 (0.2)		N = 175	N = 63	−0.3 (0.3)		N = 142	N = 63
Striatum –	N = 33	−0.8	−82.5	−59	N = 30	−0.9	−149.4	−106.7
volume MRI	−117.9 (144.1)		N = 261	N = 94	−213.4 (227.7)		N = 199	N = 72
Putamen –	N = 33	−0.6	−42.3	−30.2	N = 30	−0.9	−91.1	−65.1
volume MRI	−60.4 (103.7)		N = 516	N = 186	−130.2 (148.1)		N = 226	N = 82
Cerebellum white matter – volume MRI	N = 32	−0.99	−294.4	−210.3	N = 30	−1.3	−452.5	−323.2
	−420.5 (422.9)		N = 177	N = 64	−646.4 (485.6)		N = 99	N = 36
Cerebellum gray matter – volume MRI	N = 33	−0.8	−516.5	−369	N = 30	−1.4	−1.027.4	−733.9
	−737.9 (906.7)		N = 264	N = 95	−1467.7 (1055)		N = 91	N = 33
PONS – volume MRI	N = 33	−1.4	−252	−180	N = 30	−1.5	−459.6	−328.3
	−360 (258.5)		N = 90	N = 33	−656.5 (428.1)		N = 75	N = 27
Whole brainstem – volume MRI	N = 33	−1.3	−327.3	−233.8	N = 30	−1.6	−596.3	−425.9
	−467.5 (361.6)		N = 105	N = 38	−851.8 (546.2)		N = 72	N = 26

M0 = baseline; M6 = month 6; M12 = month 12; MRI = magnetic resonance imaging; SD = standard deviation; UMSARS = Unified Multiple System Atrophy Rating Scale.

^a^ N corresponds to the number of patients with calculated delta between M0 and M6.

^b^ Corresponds to the observed data in Aspire‐MSA: mean (SD) of the delta between M0 and M6.

^c^ Standardized effect sizes were calculated dividing the annual mean change by the SD of the annual mean change in each of the parameters studied.

^d^ Hypothetical mean si 30% reduction in the other group.

^e^ Power calculations were based on a 2‐sided significance level (α) of 5%, and a power (1‐β) of 80%, assuming hypothetical 30% (or 50%) reductions in another group.

## Discussion

This longitudinal, multicenter, case–control observational study simultaneously and prospectively monitored patients with early‐stage MSA within the same cohort. It assessed a comprehensive set of clinical, imaging, and fluid markers of disease progression over 6‐ and 12‐month follow‐up periods. Our results show that significant changes were already detected in a timeframe as short as 6 months, with further aggravation at 12 months, in line with the known aggressiveness of the disease. At baseline, neuro‐imaging markers (MRI brainstem and pons atrophy and striatal DaT‐SPECT SBR) predicted subsequent clinical aggravation (total UMSARS‐I + II score). Distinct trajectories of neuroimaging progression markers were also observed when comparing patients with MSA‐P versus patients with MSA‐C, the former presenting a greater striatal DaT‐SPECT signal reduction reflective of dopaminergic degeneration, and the latter a greater atrophy in the cerebellum (gray and white matter) and pons. Patients with higher baseline plasma NfL values had more severe disability (total UMSARS‐I + II scores), more severe dopaminergic degeneration, and a greater risk of death within 24 months after enrollment. Early dropouts had significantly higher baseline plasma NfL levels and worse QOL than completers.

### Clinical Findings

As expected, a significant worsening of the total UMSARS‐I + II score was observed at M6 (mean increase = +6.5 points) and at M12 (mean increase = +10.9 points), consistent with the magnitude of changes previously reported in other cohorts.^2^, ^10^, ^27^, ^28^ Fourteen of 34 patients exhibited a slow progression (< 10 units, as based on our median crude delta, which is well above the 3.5 points considered as minimally clinically important decline^29^) at M12, but no clinical neither neuroimaging baseline biomarkers could differentiate slow versus faster progressor, with the exception of a worse QOL and higher depression score.

Of note, we observed a significant deterioration of the MSA‐QOL score even after 6 months, whereas MoCA, BDI, and COMPASS‐31 scores did not change over time. These findings highlight the potential importance of QOL scores to track progression. Indeed, QOL longitudinal changes merit further investigation as there are no data on a short frame follow‐up of 6 months and a previous cohort of patients with early MSA found a slower progression over about 1‐year follow‐up (mean 4.3 ± 9.3, 0.9 ± 7.1, and 1.6 ± 10.6 for the motor, non‐motor, and emotional domain).^30^

### MRI Findings

It is already known that a significant worsening of MRI T1‐3D markers of brain atrophy can be detected within a year in patients with MSA.^10^, ^28^, ^31^, ^32^ Our findings demonstrated that a significant volume loss was already detectable as early as at 6 months (−4.4% and −3.2% for cerebellar white matter and pons, respectively), which is of interest for future assessments in clinical trials. The ASPIRE cohort also confirmed a significant annual volume loss of −7% and −5.8% in patients with MSA‐P + C for cerebellar white matter and pons, which were in line with previous reports (range = −6.7%–4.7%) for MSA‐P + C.^10^, ^11^ We also observed, after the 1‐year follow‐up, different patterns of atrophy between MSA‐P and MSA‐C subtypes, with a significantly greater volume loss in the cerebellar white matter and pons of patients with MSA‐C than patients with MSA‐P (−9.5% and −8.7% in patients with MSA‐C vs −5% and −3.6% in patients with MSA‐P). This is consistent with another MSA cohort using the same FreeSurfer's image analysis pipeline (annual loss of −10.1% in patients with MSA‐C vs −2.6% in patients with MSA‐P, considering the cerebellar white matter, which was the most affected region).^10^ Conversely, a higher rate of putamen atrophy at 1 year has previously been reported in patients with MSA‐P compared with patients with MSA‐C.^10^ In the ASPIRE cohort, we did not observe such a difference. Instead, the cohort showed a later onset of putamen atrophy, appearing between M6 and M12, rather than between M0 and M6. Distinct chronological and topographical patterns of MRI markers of brain atrophy in MSA may offer valuable insights into the disease's progression, potentially informing stratification strategies for future clinical trials. However, this should be further explored in larger populations, considering the limited sample size of the currently available data. The fact that the ASPIRE cohort collected MRI data in patients with MSA and matched HCs strengthens our findings, as age significantly impacts brain volume over time.

Patients with MSA‐P + C also showed a greater increase in MD than the HCs in the putamen and cerebellum white and gray matter at M6, whereas, surprisingly, such changes were no more visible at M12, being no longer observed in the cerebellum white matter. It is possible that this observation might result from an attrition bias, as the most severely affected patients may have dropped out prematurely and missed the M12 visit. It is also possible that an initial trend in MD increase might diminish at a later stage of the disease. Diffusion abnormalities have already been reported in patients with MSA‐P + C, both in the white (cerebellum and pons) and gray matter (cerebellum and putamen)^13^, ^33^ and some correlations have been reported between abnormal putamina diffusivity and motor disability in small samples of patients with MSA‐P.^31^, ^34^, ^35^, ^36^ Altogether, these preliminary data underline the potential interest of changes in MD markers for MSA, especially in the early stages, as they might precede indices of atrophy. Conversely, our findings suggest that FA markers might be less useful, as we observed great overlap between patients with MSA‐P + C and HCs at all visits, both in the cerebellum and whole brain white matter. Microstructural brain damage due to tissue degeneration is generally associated with lower FA values, but the variability of FA markers is greater than that of MD, thus reducing its sensitivity to change.^37^

Of note, MRI markers predicted clinical aggravation only in a short timeframe of 6 months, when adjusted for phenotype. There has been a previous report on specific structural baseline MRI features associated with a more rapid decline.^38^ However, in this study, a group of patients with MSA‐P with structural MRI changes was compared with a group without such changes, whereas all our patients showed baseline MRI abnormalities.

### DaT‐SPECT Scan Findings

There are only few longitudinal studies assessing DaT‐SPECT changes in small numbers of patients with MSA.^14^, ^39^ Conversely, in Parkinson's disease (PD) neuroprotective trials it is widely used to improve diagnostic accuracy at patients’ inclusion and it is frequently used as secondary/exploratory outcome especially in an early PD stage, even if evidence of its use as surrogate of neuroprotection are still debated.^40^, ^41^

We observed a significantly greater annual decrease in SBR in the striatum of patients with MSA‐P + C versus the HCs (−17.2% vs −1%, respectively, for the whole striatum), and a significant reduction in patients with MSA‐P + C from baseline to both 6‐ and 12‐months (−11.9% and −17.2%, respectively). This suggests that quantifying DaT images with DaTsoft3D software may offer a useful way to assess dopaminergic neuronal loss and disease progression over time in MSA, like in PD. We also observed that the putamen/caudate ratio was lower in patients with MSA compared with HCs at all 3 visits, indicating a sustained preferential signal reduction in the putamen over the caudate, consistent with previous findings.^14^ Interestingly, a greater SBR percentage reduction was observed in the ASPIRE cohort in patients with MSA‐P than patients with MSA‐C at both M6 (−15.4% vs −6.6%) and M12 (−24.7% vs −5.4%), whereas this was not observed in a smaller study.^39^ Indeed, the onset of signal reduction appeared earlier in patients with MSA‐P than in patients with MSA‐C, who had a relatively preserved SBR at disease onset,^39^ as happens in 5 of our patients with MSA‐C, whose putamen volume fell within normal range (data not shown).

Of note, regression models examining associations between the neuroimaging and NfL data at M0 and clinical progression over time as assessed by the total UMSARS‐I + II score revealed that DaTscan‐derived striatal SBR values at M0 significantly predicted clinical progression of the total UMSARS‐I + II score from 0 to 12 months (only for the unadjusted model), but not from 0 to 6 months (in both the adjusted and unadjusted models). However, as 5 patients with MSA‐C had a normal DaT scan, this may have reduced the power of such analyses. Notably, normal DaTscan findings are not uncommon and have been reported in approximately 25% to 30% of patients with MSA‐C.^42^, ^43^, ^44^ This possibility should be considered when including DaTscan as biomarker of progression in MSA studies.

### NfL Findings

Increasing evidence suggests that fluid biomarkers, such as NfL concentrations in CSF or plasma, are elevated in MSA, like in other neurological disorders inducing axonal damage.^18^, ^19^, ^45^ Their role in predicting disease progression remains inconsistent,^18^, ^45^ although recent evidence suggested that higher baseline concentrations were related to faster progression and fatal events.^17^ We did not assess NfLs in the HC group, but the baseline values in the patients with MSA‐P + C were higher than what is usually reported in the HC samples. We did not observe in our patients with MSA a significant increase in plasma NfL levels over time, possibly due to the large dispersion of the values and the limited number of subjects, although this should be further explored in larger and longer cohorts. On the opposite side, we observed that NfL may prove of use as a baseline biomarker of disease severity and predictor of subsequent premature dropout rate and fatal outcome.^17^ In ASPIRE, the patients who dropped out prematurely had indeed significantly higher baseline NfL levels than completers. Moreover, at baseline, the patients with higher NfL values also had higher clinical disability (UMSARS‐I + II), more severe dopaminergic degeneration (DaT‐SPECT), and a shorter survival after 18 months following enrollment.

### Implications for Clinical Trials

The ASPIRE study focused on a specific subset of patients with early MSA—those within 5 years of symptom onset and without major impairments in speech, gait, or falls—who are the most likely candidates for future disease‐modifying trials (see inclusion criteria for NCT06568237, NCT05526391, and NCT06706622). However, identifying early‐stage MSA remains challenging due to overlapping clinical features, particularly between patients with MSA‐P and patients with PD, and between patients with MSA‐C and other forms of ataxia. Consequently, large MSA cohorts often include patients with a mean disease duration of 4.5 to 5.5 years at study entry, largely due to diagnostic delays.^2^, ^27^ The strict application of the current diagnostic criteria,^46^ which include a comprehensive list of motor and non‐motor supportive features, as well as specific definitions for levodopa responsiveness and MRI markers, may have the possibility to enhance early diagnostic accuracy. Indeed, their use has been associated with high diagnostic specificity, particularly when accompanied by at least 2 supporting clinical features.^47^, ^48^ At any rate, diagnostic challenges of early MSA need to be considered for trials and observational study feasibility.

The ASPIRE results offer key insights for MSA clinical trials. MRI‐based regional volume measurements of the pons and brainstem may predict clinical worsening over 6 months, whereas DaT‐SPECT may be more reliable for 12‐month follow‐ups. However, caution is needed as some patients with MSA‐C had normal DaT‐SPECT scans at baseline. Regional volume measurements of the pons and brainstem may help optimize sample size calculations for short‐term (6 months) follow‐up studies. For annual studies, clinical and MRI markers appear to perform equally for sample size calculations. However, this assumption still requires evidence demonstrating that brain volume loss in specific regions serves as a surrogate biomarker for the perceived preservation of function in patients with MSA. Therefore, further evidence is needed to fully validate neuroimaging as a clinically meaningful biomarker for patients with MSA. Heterogeneity in the rates of progression of different biomarkers and different regions of interest between MSA sub‐types was also observed. This finding implies that to best encapsulate patient heterogeneity and inform patient selection, ideally, different neuroimaging modalities are required combined with analytical approaches encompassing a range of brain regions involved in the pathophysiology of MSA.

Additionally, NfL levels could play a critical role in patient selection, as they have been shown to predict dropouts and mortality.

Finally, due to the relatively small sample size of patients with early MSA assessed by means of 3 timepoints of follow‐up, we were not able to investigate different progression rates in clinical and neuroimaging biomarkers and possible plateau effect, possibly related to heterogeneous disease durations at inclusion. However, considering the relatively homogeneous disease duration of our sample (mean ± SD = 41.8 ± 14.9 months), it is more plausible that variations in progression rate are driven more by disease severity—such as higher NfL levels—than by disease duration. This hypothesis warrants further investigation in larger cohorts and may inform the design of future clinical trials.

### Strengths and Limitations

The ASPIRE cohort has several strengths. It included patients at an early disease stage, which is the preferred target for clinical trials, recruited from expert MSA centers to minimize misdiagnosis. Systematic assessments were conducted at 6 and 12 months, with little prior data available at 6 months. A comprehensive panel of clinical, neuroimaging, and biological markers, including DaT‐SPECT, was used, with centralized MRI data harmonization thanks to the CATI's expertise, ensuring consistency in this multicenter study.

However, limitations exist. Despite being relatively large for a rare disease, the sample size was limited, and no adjustments for multiple comparisons were made, requiring cautious interpretation. Indeed, we should consider our results more as a trend, than strong statistically supported data, that indicate to which specific brain region or biomarkers, we should perform adequately powered studies. The 1‐year follow‐up aligns with typical MSA trials, but survival data were only collected up to 24 months post‐study. No neuropathological confirmation was available. The ROI‐based MRI analysis, whereas hypothesis‐driven, may have overlooked changes in unexplored brain regions. Future studies could incorporate voxel‐based morphometry for a more comprehensive analysis.

## Conclusions

The identification of biomarkers of MSA disease progression is crucial to help optimize future trial designs in terms of patient selection and sample size, with the view of improving our ability to measure the therapeutic effects of novel treatments. They may also provide mechanistic evidence for disease modifying effects. In the ASPIRE study, we examined the trajectories and predictive value of traditional neuroimaging biomarkers derived from MRI and DaT‐SPECT, alongside plasma NfL, which has garnered increasing attention in recent years.

Our findings suggest clinical progression over a short 6‐month timeframe could be best predicted by brainstem and pons baseline atrophy. Over a 12‐month follow‐up, baseline DaT‐SPECT emerged as a better predictor. However, this should be interpreted with caution, as a subset of patients with MSA‐C showed normal DaT‐SPECT scans at baseline. Indeed, when the analysis was adjusted for MSA phenotype values only showed a trend, not statistically significant. We also observed significant heterogeneity in progression rates of various biomarkers and MRI regions of interest across MSA subtypes, highlighting the potential of these biomarkers to characterize patients based on underlying pathophysiology. This supports a data‐driven stratification approach, reducing between‐patient variability and suggesting the use of multiple neuroimaging modalities alongside analytical techniques that capture the diversity of brain regions involved in MSA.

Plasma NfL levels are associated with greater baseline clinical impairment and mortality rate and could aid with patient selection in future trials. On a related note, once specific brain volume will be demonstrated as valuable surrogate biomarkers of MSA disease progression, they could be adopted to optimize sample size calculation in short‐term clinical trials.

In conclusion, this observational study highlights neuroimaging changes occurring within a 6‐month timeframe in patients with early MSA. It also emphasizes the prognostic importance of elevated NfL levels in predicting mortality and study dropout. Potential distinct progression patterns for MSA‐P and MSA‐C subtypes are observed, suggesting the relevance of a multimodal assessment, including different biomarkers.

## Author Contributions

A.D., H.C., C.T., S.T., C.T., F.D., A.M., A.E., L.D., A.F., A.G.C., S.F., A.F.S., B.P., and A.P.L.T. contributed to the acquisition of data and data analysis. A.S., J.C.C., and P.P. contributed to the conception and design of the study. M.F., N.d.C., W.M., O.R., A.S., P.P., P.G., and V.R. contributed to drafting the text or preparing the figures.

## Potential Conflicts of Interest

The authors report no conflict of interest.

## Supporting information


**Supplementary Figure S1.** Cross‐sectional correlations with total UMSARS‐I + II in all MSA‐ (P + C) patients.

**Supplementary Figure S2.** Longitudinal correlations with total UMSARS‐I + II in all MSA‐ (P + C) patients.

**Supplementary Figure S3.** Cross‐sectional correlations with MSA‐QoL in all MSA‐ (P + C) patients.

**Supplementary Figure S4.** Longitudinal correlations with MSA‐QoL in all patients with MSA‐ (P + C).

**Supplementary Figure S5.** Supplementary Figure 5.

**Supplementary Tables S1–S6.** S1: Baseline biomarkers comparisons;
S2a. Percent change with 95% CI for clinical, neuroimaging and wet biomarkers for MSA – (P + C).
S2b. Percent change with 95% CI for clinical, neuroimaging and wet biomarkers for MSA‐P and MSA‐C, separately.
S3: Biomarkers progression (percentage changes) for patients with MSA‐P and MSA‐P, separately. MWW = *p*‐value of the Mann‐–Whitney‐Wilcoxon test. Adjusted model = *p*‐value of the interaction of the mixed linear regression model for repeated measures: response variable was the crude values and explanatory variables were the group (MSA‐P/MSA‐C), the visit (M0, M6, and M12) and the interaction group * visit. Each model was adjusted for age and sex.
S4: Spearman Correlation matrix for total UMSARS‐I + II ‐ At M0 ‐ MSA‐ (P + C) Patients. CSF NfL values.
S5: Cross‐sectional correlations at baseline for MSA‐(P + C) patients.
S5: Baseline clinical, neuroimaging, and wet biomarkers comparisons for slow vs. fast progressors and dropout (missing patients at M12) vs. all the others.
S6: Clinical and biomarkers details of the 5 MSA‐C patients with normal Dat‐Scan.

## Data Availability

Anonymized data from this study will be available from the corresponding author upon reasonable request from any qualified researcher, following the EU General Data Protection Regulation. The study protocol and statistical analysis plan will be shared upon request.
