# Supplementary figures and images for: Disease Progression in Multiple System Atrophy: The ASPIRE Multi‐Modal Biomarker Study
Source: Ann Neurol. 2025 Aug 26;99(1):96–113. doi: 10.1002/ana.70028 (PMC12946593; doi:10.1002/ana.70028)

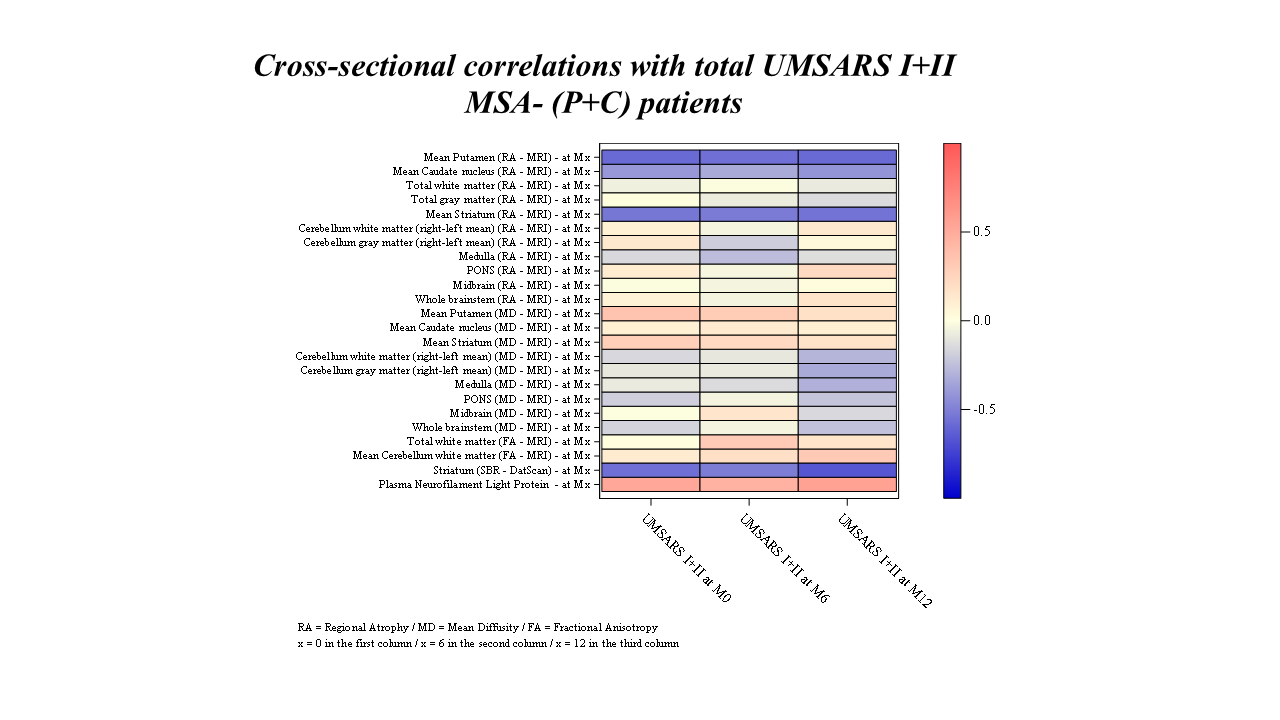

Supplement: Supplementary file 2 — Supplementary Figure S2. Longitudinal correlations with total UMSARS‐I + II in all MSA‐ (P + C) patients. [file ANA-99-96-s003.tif]

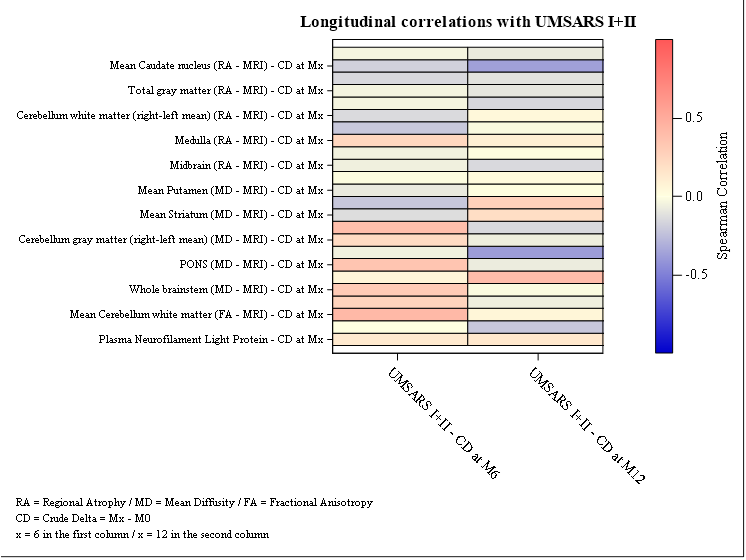

Supplement: Supplementary file 3 — Supplementary Figure S3. Cross‐sectional correlations with MSA‐QoL in all MSA‐ (P + C) patients. [file ANA-99-96-s005.tif]

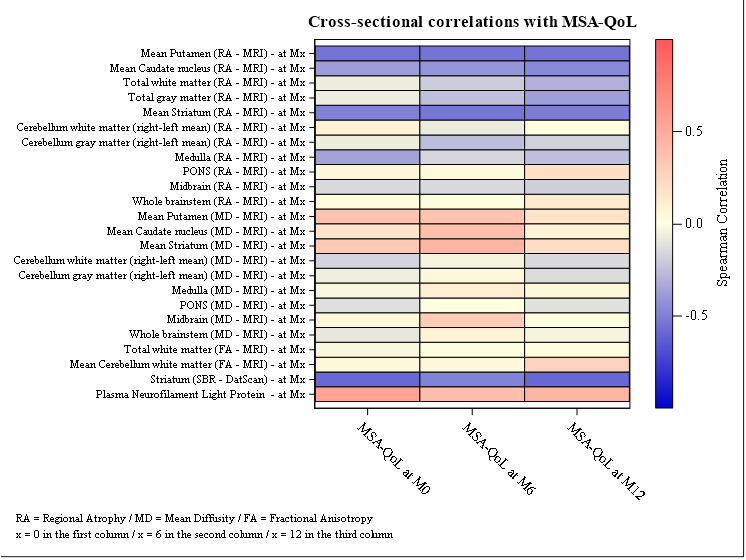

Supplement: Supplementary file 4 — Supplementary Figure S4. Longitudinal correlations with MSA‐QoL in all patients with MSA‐ (P + C). [file ANA-99-96-s001.tif]

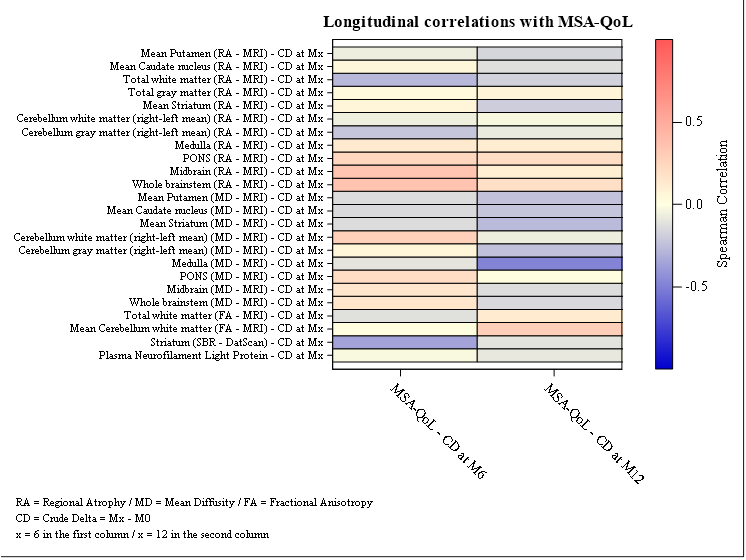

Supplement: Supplementary file 5 — Supplementary Figure S5. Supplementary Figure 5. [file ANA-99-96-s002.tif]
