# Supplementary material for: Disease Progression in Multiple System Atrophy: The ASPIRE Multi‐Modal Biomarker Study
Source: Ann Neurol. 2025 Aug 26;99(1):96–113. doi: 10.1002/ana.70028 (PMC12946593; doi:10.1002/ana.70028)
Supplement: Supplementary file 6 — Supplementary Tables S1–S6. S1: Baseline biomarkers comparisons; S2a. Percent change with 95% CI for clinical, neuroimaging and wet biomarkers for MSA – (P + C). S2b. Percent change with 95% CI for clinical, neuroimaging and wet biomarkers for MSA‐P and MSA‐C, separately. S3: Biomarkers progression (percentage changes) for patients with MSA‐P and MSA‐P, separately. MWW = p‐value of the Mann‐–Whitney‐Wilcoxon test. Adjusted model = p‐value of the interaction of the mixed linear regression model for repeated measures: response variable was the crude values and explanatory variables were the group (MSA‐P/MSA‐C), the visit (M0, M6, and M12) and the interaction group * visit. Each model was adjusted for age and sex. S4: Spearman Correlation matrix for total UMSARS‐I + II ‐ At M0 ‐ MSA‐ (P + C) Patients. CSF NfL values. S5: Cross‐sectional correlations at baseline for MSA‐(P + C) patients. S5: Baseline clinical, neuroimaging, and wet biomarkers comparisons for slow vs. fast progressors and dropout (missing patients at M12) vs. all the others. S6: Clinical and biomarkers details of the 5 MSA‐C patients with normal Dat‐Scan. [file ANA-99-96-s006.docx]

**SUPPLEMENTARY TABLES**

**Table S1. Baseline biomarkers comparisons.**

|  | **HC** | **MSA – (P+C)** | **MSA-P** | **MSA-C** | **MSA-P vs. MSA-C** | **HC vs. All MSA** |
| --- | --- | --- | --- | --- | --- | --- |
| **Characteristics at baseline** | **(N=20)** | **(N= 41)** | **(N=26)** | **(N=15)** | **SD** | **SD** |
| **MRI *- regional atrophy*** |  |  |  |  |  |  |
| Putamen | 4709.65 (688.68) | 3756.87 (1076.92) | 3350.44 (986.95) | 4434.24 (879.97) | **-1.13** | **-0.91** |
| Caudate nucleus | 3426.98 (545.09) | 3257.72 (569.57) | 3067 (532.57) | 3575.59 (494.24) | **-0.95** | **-0.28** |
| Total white matter | 442309.75 (67183.02) | 445314.45 (49908.42) | 439587.82 (47952.29) | 454858.83 (53298.85) | **-0.3** | -0.06 |
| Total gray matter | 591343.48 (80401.91) | 581989.83 (46584.34) | 577982.2 (39747.1) | 588669.23 (57100.93) | **-0.35** | **-0.19** |
| Mean Striatum | 8136.63 (1163.64) | 7014.59 (1598.88) | 6417.44 (1462.51) | 8009.83 (1325.38) | **-1.16** | **-0.76** |
| Cerebellum white matter | 13382.37 (1465.9) | 10662.92 (3330.16) | 12362.09 (2485.88) | 7830.97 (2559.58) | **1.67** | **-0.93** |
| Cerebellum gray matter | 50411.62 (5399.87) | 46802.53 (6349.76) | 48901.66 (5044.49) | 43303.99 (6907.44) | **0.88** | **-0.49** |
| Medulla | 4902.59 (440.72) | 4744.15 (470.22) | 4743.76 (467.69) | 4744.81 (490.88) | -0.03 | **-0.29** |
| Pons | 14741.6 (1773.47) | 12651.28 (3096.68) | 14065.01 (2388.23) | 10295.06 (2723.67) | **1.52** | **-0.78** |
| Midbrain | 6007.85 (628.91) | 5783.34 (663.6) | 5842.83 (590.87) | 5684.2 (781.91) | **0.25** | **-0.34** |
| Brainstem | 25900.05 (2720.04) | 23397.2 (3936.83) | 24889.53 (3292.31) | 20909.97 (3740.06) | **1.22** | **-0.74** |
| **MRI- mean diffusivity** |  |  |  |  |  |  |
| Putamen | 0.75 (0.04) | 0.79 (0.1) | 0.82 (0.1) | 0.74 (0.05) | **1.09** | **0.24** |
| Caudate nucleus | 0.95 (0.09) | 0.98 (0.1) | 1 (0.11) | 0.95 (0.09) | **0.46** | **0.22** |
| Striatum | 1.7 (0.13) | 1.77 (0.17) | 1.82 (0.18) | 1.68 (0.13) | **0.81** | **0.39** |
| Cerebellum white matter | 0.74 (0.05) | 0.83 (0.12) | 0.79 (0.1) | 0.92 (0.1) | **-1.58** | **0.96** |
| Cerebellum gray matter | 0.89 (0.07) | 0.99 (0.16) | 0.93 (0.12) | 1.1 (0.17) | **-1.28** | **0.77** |
| Medulla | 0.96 (0.12) | 1.04 (0.15) | 0.99 (0.13) | 1.11 (0.15) | **-0.89** | **0.58** |
| Pons | 0.95 (0.12) | 0.99 (0.17) | 0.95 (0.17) | 1.06 (0.15) | **-0.69** | **0.29** |
| Midbrain | 0.89 (0.09) | 0.94 (0.08) | 0.95 (0.08) | 0.91 (0.09) | **0.55** | **0.56** |
| Brainstem | 0.94 (0.11) | 0.99 (0.13) | 0.96 (0.13) | 1.03 (0.12) | **-0.55** | **0.44** |
| **MRI- fractional anisotropy** |  |  |  |  |  |  |
| Total white matter | 0.32 (0.03) | 0.32 (0.03) | 0.33 (0.03) | 0.32 (0.03) | 0.16 | **0.29** |
| Cerebellar white matter | 0.35 (0.03) | 0.33 (0.08) | 0.35 (0.09) | 0.29 (0.04) | **1.21** | **-0.63** |
| **Dat-Scan** |  |  |  |  |  |  |
| caudate nucleus – SBR | 4.53 (1.03) | 2.75 (1.29) | 2.34 (1.08) | 3.47 (1.34) | **-0.87** | **-1.44** |
| putamen– SBR | 3.91 (0.85) | 1.49 (1.1) | 0.96 (0.64) | 2.4 (1.15) | **-1.39** | **-2.26** |
| Striatum | 4.18 (0.9) | 2.04 (1.14) | 1.57 (0.77) | 2.87 (1.22) | **-1.21** | **-2.06** |
| Putamen vs. caudate ratio | 0.87 (0.1) | 0.5 (0.19) | 0.41 (0.17) | 0.66 (0.13) | **-1.61** | **-2.66** |

**Table S2a. Percent change with 95% CI f****or clinical, neuroimaging and wet biomarkers for MSA – (P+C).**

|  |  |  |  |  |  |  |  |  |  |  |  |  |
| --- | --- | --- | --- | --- | --- | --- | --- | --- | --- | --- | --- | --- |
|  | **HC** | | | | | | **MSA (P+C)** | | | | | |
| **Characteristics** | **M0-M6** | | **M0-M12** | | **M6-M12** | | **M0-M6** | | **M0-M12** | | **M6-M12** | |
|  | **Mean (SD)** | **95% CI** | **Mean (SD)** | **95% CI** | **Mean (SD)** | **95% CI** | **Mean (SD)** | **95% CI** | **Mean (SD)** | **95% CI** | **Mean (SD)** | **95% CI** |
| **MSA-QoL** |  |  |  |  |  |  |  |  |  |  |  |  |
| *Total Score* | NA | NA | NA | NA | NA | NA | 21.5 (42.2) | **7;36** | 31.6 (36) | **19;44.1** | 13.1 (32) | **1.5;24.6** |
| *Motor sub-score* | NA | NA | NA | NA | NA | NA | 30.3 (48.2) | **13.7;46.8** | 51.6 (55.1) | **32.4;70.8** | 22.1 (45.2) | **5.8;38.4** |
| *Non-motor sub-score* | NA | NA | NA | NA | NA | NA | 21.3 (45.8) | **5.6;37** | 23.4 (32.5) | **12.1;34.8** | 14.9 (52.1) | -3.9;33.7 |
| *Emotional subs-core* | NA | NA | NA | NA | NA | NA | 30.6 (85.1) | **1.4;59.8** | 36.1 (89.5) | **4.9;67.4** | 8.9 (52.3) | -10;27.8 |
| **Moca** | NA | NA | NA | NA | NA | NA | -2.1 (10.1) | -5.8;1.5 | -3 (10.2) | -6.8;0.7 | -1.3 (11.1) | -5.5;2.9 |
| **BDI** | NA | NA | NA | NA | NA | NA | 12.1 (48) | -5;29.1 | 21.3 (53.4) | **2.1;40.5** | 15.2 (53.2) | -5.1;35.4 |
| **Compass-31** |  |  |  |  |  |  |  |  |  |  |  |  |
| *Total score* | NA | NA | NA | NA | NA | NA | 78.7 (315.1) | -29.6;186.9 | 25 (86) | -5.5;55.5 | 14 (61) | -8.4;36.3 |
| *Bladder sub-score* | NA | NA | NA | NA | NA | NA | 35.8 (79.3) | **7.2;64.4** | 51.5 (91.2) | **18.1;85** | 26.5 (70.9) | -0.5;53.5 |
| *Gastrointestinal sub-score* | NA | NA | NA | NA | NA | NA | 21.9 (79) | -6.1;49.9 | 18.4 (71.8) | -7.9;44.7 | 11.5 (101.2) | -26.9;50 |
| *Orthostatic intolerance sub-score* | NA | NA | NA | NA | NA | NA | -29.4 (53.2) | **-52.4;-6.3** | -30 (51.2) | **-52.2;-7.8** | -38.9 (48.5) | **-63;-14.8** |
| *Pupillomotor sub-score* | NA | NA | NA | NA | NA | NA | 30.3 (59.2) | **8.6;52.1** | 35.5 (77) | **6.2;64.8** | 6.4 (51.9) | -13.3;26.2 |
| *Secretomotor sub-score* | NA | NA | NA | NA | NA | NA | 27.5 (84.2) | -5.8;60.8 | 6 (125.8) | -44.9;56.8 | -9.2 (64) | -35;16.7 |
| *Vasomotor sub-score* | NA | NA | NA | NA | NA | NA | -50 (57.7) | -141.9;41.9 | -13.3 (50.6) | -76.1;49.4 | 2.2 (27.2) | -26.3;30.8 |
| **Clinical** |  |  |  |  |  |  |  |  |  |  |  |  |
| UMSARS |  |  |  |  |  |  |  |  |  |  |  |  |
| *I* | NA | NA | NA | NA | NA | NA | 22.7 (22.7) | **14.9;30.5** | 34.8 (21.6) | **27.3;42.3** | 14.7 (17.8) | **8.3;21.1** |
| *II* | NA | NA | NA | NA | NA | NA | 18.5 (20.7) | **11.4;25.6** | 29.2 (24.6) | **20.6;37.7** | 10.9 (22.2) | **2.9;18.9** |
| *I+II* | NA | NA | NA | NA | NA | NA | 19.8 (19) | **13.3;26.4** | 31.1 (17.7) | **24.9;37.2** | 12.3 (15.2) | **6.8;17.7** |
| *IV* | NA | NA | NA | NA | NA | NA | 27.1 (62.8) | **5.6;48.7** | 49.8 (61.5) | **28.6;70.9** | 24.2 (33.2) | **12.5;36** |
| MSA-Qol, total | NA | NA | NA | NA | NA | NA | 21.5 (42.2) | **1.4;59.8** | 31.6 (36) | **4.9;67.4** | 13.1 (32) | -10;27.8 |
| *Motor* | NA | NA | NA | NA | NA | NA | 30.3 (48.2) | **13.7;46.8** | 51.6 (55.1) | **32.4;70.8** | 22.1 (45.2) | **5.8;38.4** |
| *Non-motor* | NA | NA | NA | NA | NA | NA | 21.3 (45.8) | **5.6;37** | 23.4 (32.5) | **12.1;34.8** | 14.9 (52.1) | -3.9;33.6 |
| E*motional* | NA | NA | NA | NA | NA | NA | 30.6 (85.1) | **7;36** | 36.1 (89.5) | **19;44.1** | 8.9 (52.3) | **1.5;24.6** |
| **MRI *- regional atrophy*** |  |  |  |  |  |  |  |  |  |  |  |  |
| Putamen | -0.6 (1.9) | -1.5;0.4 | -0.6 (1.6) | -1.4;0.2 | 0 (1.1) | -0.6;0.5 | -1.5 (2.5) | **-2.4;-0.7** | -3.3 (3.9) | **-4.8;-1.9** | -1.7 (2.9) | **-2.8;-0.6** |
| Caudate nucleus | -0.5 (1.6) | -1.3;0.3 | -0.6 (1) | **-1.1;-0.1** | -0.1 (1.3) | -0.7;0.6 | -1.9 (2.8) | **-2.9;-0.9** | -2.9 (4.7) | **-4.6;-1.2** | -1.1 (3.5) | -2.4;0.2 |
| Total white matter | -0.1 (1) | -0.6;0.4 | -0.5 (1) | -1;0 | -0.4 (1) | -0.9;0.1 | -0.6 (1.4) | **-1.1;-0.1** | -1.1 (1.3) | **-1.6;-0.6** | -0.7 (1.2) | **-1.2;-0.3** |
| Total gray matter | 0 (1.7) | -0.9;0.8 | -0.4 (1.2) | -1;0.2 | -0.3 (1.2) | -0.9;0.2 | -1 (1.8) | **-1.6;-0.3** | -1.6 (1.6) | **-2.2;-1** | -0.7 (1.1) | **-1.1;-0.2** |
| Mean Striatum | -0.5 (1.7) | -1.4;0.3 | -0.6 (1.2) | -1.2;0 | 0 (0.9) | -0.5;0.4 | -1.7 (2.2) | **-2.5;-1** | -3.2 (3.6) | **-4.5;-1.9** | -1.5 (2.7) | **-2.5;-0.4** |
| Cerebellum white matter | -0.4 (2.7) | -1.8;0.9 | -1 (2.1) | -2.1;0 | -0.6 (2.3) | -1.7;0.6 | -4.4 (4.3) | **-5.9;-2.8** | -7 (5.5) | **-9;-4.9** | -3.4 (4.6) | **-5.1;-1.6** |
| Cerebellum gray matter | 0.7 (1.8) | -0.2;1.6 | -0.1 (1.5) | -0.8;0.7 | -0.7 (0.9) | **-1.2;-0.3** | -1.6 (2.1) | **-2.4;-0.9** | -3.3 (2.4) | **-4.2;-2.4** | -1.6 (2.1) | **-2.4;-0.8** |
| Medulla | 0.3 (3.6) | -1.5;2.1 | 0.1 (2.9) | -1.3;1.5 | -0.2 (2.9) | -1.6;1.3 | -0.7 (3.6) | -2;0.6 | -1.6 (2.6) | **-2.5;-0.6** | -0.5 (2.6) | -1.5;0.5 |
| Pons | -0.4 (1.4) | -1;0.3 | -0.5 (1.7) | -1.3;0.4 | -0.1 (1.1) | -0.6;0.4 | -3.2 (2.3) | **-4;-2.4** | -5.8 (3.7) | **-7.2;-4.4** | -2.9 (2) | **-3.6;-2.1** |
| Midbrain | -0.5 (1.8) | -1.4;0.4 | -0.9 (1.7) | -1.7;0 | -0.3 (1.5) | -1.1;0.4 | -1.1 (2.8) | **-2;-0.1** | -2 (2.4) | **-2.8;-1.1** | -1.3 (2.2) | **-2.1;-0.4** |
| Brainstem | -0.3 (1.6) | -1.1;0.5 | -0.5 (1.8) | -1.4;0.4 | -0.2 (1.2) | -0.8;0.4 | -2.1 (1.6) | **-2.7;-1.5** | -3.8 (2.4) | **-4.7;-2.9** | -1.9 (1.5) | **-2.5;-1.3** |
| **MRI- mean diffusivity** |  |  |  |  |  |  |  |  |  |  |  |  |
| Putamen | 0.2 (2.7) | -1.2;1.5 | 0.6 (2.4) | -0.6;1.7 | 0.7 (2.9) | -0.8;2.2 | 2.1 (2.6) | **1.1;3** | 2.2 (3.8) | **0.6;3.7** | 0.7 (3.1) | -0.5;2 |
| Caudate nucleus | 0.5 (3.4) | -1.2;2.2 | 1.8 (4) | -0.4;4 | 1.7 (4.2) | -0.6;4.1 | 2.7 (4.9) | **1;4.5** | 5.3 (7.9) | **2.2;8.5** | 2.9 (7.1) | -0.1;5.8 |
| Striatum | 0.6 (2.1) | -0.5;1.6 | 1.2 (2.5) | -0.1;2.6 | 1 (2.8) | -0.6;2.6 | 2.4 (3.5) | **1.1;3.7** | 3.7 (5.3) | **1.4;5.9** | 1.8 (4.5) | -0.2;3.7 |
| Cerebellum white matter | 0.7 (2.7) | -0.7;2 | 0.6 (3.4) | -1.1;2.3 | -0.1 (3) | -1.5;1.5 | 2 (3.4) | **0.8;3.2** | 0.7 (4.7) | -1.2;2.6 | -0.4 (3.8) | -2;1.3 |
| Cerebellum gray matter | 1.2 (2.5) | 0;2.4 | 1 (3.7) | -0.8;2.8 | -0.2 (3.5) | -1.9;1.6 | 2.5 (3.6) | **1.2;3.8** | 2.4 (4.3) | **0.7;4.1** | 0.4 (3.6) | -1.1;1.8 |
| Medulla | 0.7 (5.3) | -2;3.3 | 3.3 (5.2) | **0.7;6** | 3.1 (4.7) | **0.7;5.5** | 2.2 (6.9) | -0.5;4.8 | 2.1 (7.8) | **-1.2;5.3** | 0.5 (5.8) | -2.1;3 |
| Pons | 0.7 (5.8) | -2.3;3.7 | 1.5 (4.6) | -0.8;3.7 | 0.7 (5.6) | -2.2;3.6 | 1.3 (5.8) | -0.8;3.4 | 1.4 (6.5) | -1.2;3.9 | 1.5 (5.1) | -0.6;3.6 |
| Midbrain | 1.1 (4.6) | -1.2;3.4 | 1.7 (4.5) | -0.5;4 | 0.7 (3.9) | -1.2;2.6 | 0.8 (4.2) | -0.7;2.3 | 2.4 (4.4) | **0.7;4.1** | 1.9 (3.9) | **0.4;3.4** |
| Brainstem | 0.4 (4.9) | -2;2.8 | 2.3 (3.3) | **0.7;3.9** | 2 (5) | -0.4;4.5 | 1.3 (4.9) | -0.5;3 | 1.2 (4.7) | -0.7;3 | 0.8 (4.7) | -1.1;2.6 |
| **MRI- fractional anisotropy** |  |  |  |  |  |  |  |  |  |  |  |  |
| Total white matter | -1.3 (3.3) | -2.9;0.3 | -0.8 (5.8) | -3.6;2.1 | 0.6 (6.2) | -2.5;3.7 | -1.7 (2.9) | **-2.8;-0.7** | -0.6 (7.5) | -3.5;2.3 | 1 (7) | -1.8;3.8 |
| Cerebellar white matter | -1.3 (3.4) | -3;0.4 | -1 (6.1) | -4.1;2 | 0.3 (5.7) | -2.5;3.1 | -2.3 (4.4) | **-4;-0.6** | -2.7 (6) | **-5.2;-0.2** | -0.5 (6) | -3.2;2.3 |
| **Dat-Scan** |  |  |  |  |  |  |  |  |  |  |  |  |
| caudate nucleus – SBR | NA | NA | 0.8 (13.2) | -5.9;7.6 | NA | NA | -11 (14.6) | **-16;-6** | -14 (22.7) | **-22.3;-5.7** | -3.5 (24) | -12.4;5.5 |
| putamen– SBR | NA | NA | -2.5 (13.8) | -9.6;4.6 | NA | NA | -8.8 (24.5) | **-17.2;-0.4** | -19.3 (21.3) | **-27.1;-11.5** | -11.5 (19.1) | **-18.6;-4.3** |
| Striatum | NA | NA | -1 (11.4) | -6.8;4.9 | NA | NA | -11.9 (11.5) | **-15.8;-7.9** | -17.2 (18.6) | **-24;-10.4** | -6.9 (18.9) | -14;0.2 |
| Putamen vs. caudate ratio | NA | NA | -2.6 (12.9) | -9.2;4 | NA | NA | 7.2 (43.9) | -7.9;22.3 | 3.5 (63.7) | -19.9;26.8 | -2.7 (35.6) | -16;10.6 |
| **Wet biomarkers** |  |  |  |  |  |  |  |  |  |  |  |  |
| Plasma Neurofilament Light chain | NA | NA | NA | NA | NA | NA | 8.9 (22.9) | 0.9;16.9 | 19 (41.4) | 4.3;33.7 | 9.5 (30.9) | -1.9;20.8 |

**Table S2b. Percent change with 95% CI for clinical, neuroimaging and wet biomarkers for MSA-P and MSA-C, separately.**

|  |  |  |  |  |  |  |  |  |  |  |  |  |
| --- | --- | --- | --- | --- | --- | --- | --- | --- | --- | --- | --- | --- |
|  | **MSA C** | | | | | | **MSA P** | | | | | |
| **Characteristics** | **M0-M6** | | **M0-M12** | | **M6-M12** | | **M0-M6** | | **M0-M12** | | **M6-M12** | |
|  | **Mean (SD)** | **95% CI** | **Mean (SD)** | **95% CI** | **Mean (SD)** | **95% CI** | **Mean (SD)** | **95% CI** | **Mean (SD)** | **95% CI** | **Mean (SD)** | **95% CI** |
| **MSA-QoL** |  |  |  |  |  |  |  |  |  |  |  |  |
| *Total Score* | 22.5 (45.5) | -3.8;48.8 | 29.4 (36.3) | **7.5;51.4** | 12.5 (33.3) | -7.6;32.7 | 20.8 (41.1) | **2.1;39.5** | 32.9 (36.6) | **16.2;49.6** | 13.5 (32) | -2;28.9 |
| *Motor sub-score* | 25.2 (36.7) | **4;46.4** | 43.1 (32) | **23.8;62.4** | 25.2 (59.1) | -10.5;60.9 | 33.7 (55.2) | **8.6;58.8** | 56.9 (65.7) | **27;86.8** | 20 (34.2) | **3.5;36.5** |
| *Non-motor sub-score* | 28.5 (48.3) | **0.6;56.4** | 28.3 (36) | **6.6;50.1** | 8.4 (37.6) | -14.3;31.1 | 16.5 (44.5) | -3.8;36.8 | 20.4 (30.7) | **6.4;34.4** | 19.3 (60.7) | -10;48.5 |
| *Emotional subs-core* | 40.4 (112.2) | -24.4;105.2 | 47.2 (122.7) | -26.9;121.4 | 5.9 (31.7) | -13.2;25.1 | 24.1 (63.4) | -4.8;52.9 | 29.3 (63.6) | **0.3;58.2** | 10.9 (63.6) | -19.7;41.6 |
| **Moca** | -2.4 (9.4) | -8;3.3 | -6.1 (9.8) | -12.3;0.1 | -5.3 (10.5) | -11.9;1.4 | -2 (10.9) | -7.2;3.3 | -1.1 (10.2) | -6;3.8 | 1.5 (10.9) | -4.1;7.1 |
| **BDI** | 19 (58.4) | -16.3;54.3 | 18.2 (70.3) | -24.3;60.7 | 8.8 (51.1) | -23.7;41.3 | 7.6 (40.9) | -11.6;26.7 | 23.4 (39.9) | 4.2;42.7 | 19.7 (55.8) | -9;48.3 |
| **Compass-31** |  |  |  |  |  |  |  |  |  |  |  |  |
| *Total score* | 119.8 (485.2) | -160.4;399.9 | 31 (94.8) | -26.3;88.3 | 26.3 (73.4) | -18;70.7 | 51.2 (117.4) | -2.2;104.7 | 21.1 (82.2) | -17.3;59.6 | 5 (50.6) | -20.2;30.2 |
| *Bladder sub-score* | 61.3 (88.1) | **10.5;112.2** | 71.8 (98.6) | **12.2;131.3** | 35.5 (78.5) | -11.9;82.9 | 16 (67.8) | -17.7;49.7 | 36.9 (85.3) | -5.5;79.3 | 19.2 (65.9) | -15.9;54.3 |
| *Gastrointestinal sub-score* | 33.1 (107.7) | -35.3;101.6 | 52.3 (103.9) | -17.5;122.1 | -13.6 (46.9) | -45.1;17.9 | 15.5 (59.1) | -11.5;42.4 | -0.3 (37.7) | **-17.9;17.4** | 26.9 (122.1) | -33.8;87.6 |
| *Orthostatic intolerance sub-score* | -49.6 (48.6) | **-84.4;-14.8** | -27.1 (57) | -70.9;16.7 | -43.8 (65.1) | -112;24.5 | -13.8 (53.1) | -45.9;18.3 | -31.9 (49.4) | -60.4;-3.4 | -36.5 (41) | **-62.5;-10.4** |
| *Pupillomotor sub-score* | 33.6 (38.2) | **10.5;56.7** | 40.6 (70.4) | -4.2;85.4 | 11.2 (54.9) | -21.9;44.4 | 28 (71.7) | -7.6;63.7 | 31.9 (83.3) | -10.9;74.7 | 2.5 (50.8) | -24.5;29.6 |
| *Secretomotor sub-score* | 39.2 (114) | -37.4;115.8 | 3.2 (106) | -72.6;79 | -16.3 (62.7) | -61.2;28.6 | 19.4 (58.6) | -11.9;50.6 | 7.7 (140.2) | **-67;82.4** | -4.7 (66.4) | -40.1;30.7 |
| *Vasomotor sub-score* | - | - | - | - | 4.4 (26.9) | -62.5;71.4 | -66.7 (57.7) | -210.1;76.8 | -25 (50) | -104.6;54.6 | 0 (33.3) | -82.8;82.8 |
| **Clinical** |  |  |  |  |  |  |  |  |  |  |  |  |
| UMSARS |  |  |  |  |  |  |  |  |  |  |  |  |
| *I* | 23.9 (25.4) | **9.2;38.6** | 33 (23.7) | **18.7;47.4** | 11.2 (16.3) | **1.3;21.1** | 21.9 (21.2) | **12.2;31.6** | 35.9 (20.8) | **26.5;45.4** | 17.1 (18.8) | **8.1;26.2** |
| *II* | 16.3 (19.2) | **5.2;27.4** | 23.9 (15.8) | **14.4;33.5** | 7 (15.9) | -2.6;16.6 | 20.0 (21.9) | **10;29.9** | 32.4 (28.6) | **19.4;45.4** | 13.5 (25.8) | **1.1;25.9** |
| *I+II* | 18.8 (19.4) | **7.6;30** | 27.4 (14.8) | **18.5;36.4** | 9 (12.6) | **1.3;16.6** | 20.5 (19.2) | **11.7;29.2** | 33.3 (19.2) | **24.6;42.1** | 14.5 (16.6) | **6.5;22.5** |
| *IV* | 8.3 (36.8) | -12.9;29.6 | 26.9 (43.9) | **0.4;53.4** | 20.5 (31.3) | **1.6;39.4** | 39.7 (73.5) | **6.2;73.1** | 63.3 (67.2) | **33.5;93** | 26.7 (34.9) | **10.3;43** |
| MSA-Qol, total | 22.5 (45.5) | -24.4;105.2 | 29.4 (36.3) | -26.9;121.4 | 12.5 (33.3) | -13.2;25 | 20.8 (41.1) | -4.8;52.9 | 32.9 (36.6) | **0.3;58.2** | 13.5 (32) | -19.7;41.6 |
| *Motor* | 25.2 (36.7) | **4;46.3** | 43.1 (32) | **23.8;62.4** | 25.2 (59.1) | -10.5;60.9 | 33.7 (55.2) | **8.6;58.8** | 56.9 (65.7) | **27;86.8** | 20 (34.2) | **3.5;36.5** |
| *Non-motor* | 28.5 (48.3) | **0.6;56.4** | 28.3 (36) | **6.6;50.1** | 8.4 (37.6) | -14.3;31.1 | 16.5 (44.5) | -3.8;36.8 | 20.4 (30.7) | **6.4;34.4** | 19.3 (60.7) | -10;48.5 |
| E*motional* | 40.4 (112.2) | -3.8;48.8 | 47.2 (122.7) | **7.5;51.4** | 5.9 (31.7) | -7.6;32.7 | 24.1 (63.4) | **2.1;39.5** | 29.3 (63.6) | **16.2;49.5** | 10.9 (63.6) | -1.9;28.9 |
| **MRI *- regional atrophy*** |  |  |  |  |  |  |  |  |  |  |  |  |
| Putamen | -1.6 (2.9) | -3.3;0 | -3.2 (3.4) | **-5.3;-1.1** | -1.4 (2.7) | -3;0.3 | -1.5 (2.3) | **-2.6;-0.4** | -3.5 (4.3) | **-5.6;-1.3** | -1.9 (3.2) | **-3.6;-0.2** |
| Caudate nucleus | -1.5 (1.7) | **-2.5;-0.6** | -1.2 (2) | -2.4;0 | 0.2 (2.9) | -1.5;2 | -2.2 (3.5) | **-3.9;-0.5** | -4.2 (5.7) | **-7.1;-1.3** | -2.2 (3.7) | **-4.1;-0.2** |
| Total white matter | 0.1 (1.1) | -0.6;0.7 | -0.7 (1.3) | -1.5;0.1 | -0.9 (1) | **-1.5;-0.3** | -1.1 (1.4) | **-1.8;-0.4** | -1.4 (1.3) | **-2;-0.7** | -0.6 (1.4) | -1.3;0.1 |
| Total gray matter | -0.6 (1.3) | -1.3;0.1 | -1.4 (1.4) | **-2.2;-0.6** | -0.7 (1.2) | -1.4;0 | -1.2 (2.1) | **-2.2;-0.2** | -1.7 (1.9) | **-2.7;-0.8** | -0.7 (1.1) | -1.2;-0.1 |
| Mean Striatum | -1.6 (2) | **-2.7;-0.5** | -2.3 (2.5) | **-3.8;-0.8** | -0.7 (2.6) | -2.2;0.9 | -1.8 (2.4) | **-3;-0.7** | -3.9 (4.1) | **-6;-1.8** | -2.1 (2.7) | -3.5;-0.7 |
| Cerebellum white matter | -4.3 (4.8) | **-7;-1.5** | -9.5 (5.2) | **-12.7;-6.4** | -5.4 (5.1) | **-8.4;-2.3** | -4.4 (4.1) | **-6.5;-2.4** | -5 (4.9) | **-7.5;-2.5** | -1.6 (3.3) | -3.5;0.2 |
| Cerebellum gray matter | -1.1 (2.6) | -2.7;0.4 | -4.4 (2.6) | **-6;-2.9** | -3.1 (1.4) | **-3.9;-2.2** | -2 (1.6) | **-2.7;-1.2** | -2.4 (1.9) | **-3.4;-1.5** | -0.5 (1.9) | -1.5;0.5 |
| Medulla | -1 (2.3) | -2.4;0.3 | -1.2 (1.4) | **-2.1;-0.4** | -0.2 (2) | -1.4;1 | -0.5 (4.3) | -2.5;1.6 | -1.8 (3.2) | **-3.5;-0.2** | -0.7 (3.1) | -2.4;0.9 |
| Pons | -4.6 (1.7) | **-5.6;-3.6** | -8.7 (1.8) | **-9.8;-7.6** | -4.2 (1.4) | **-5;-3.3** | -2.2 (2.2) | **-3.3;-1.2** | -3.6 (3.2) | **-5.2;-1.9** | -1.9 (1.9) | **-2.9;-0.8** |
| Midbrain | -0.7 (2.7) | -2.3;0.8 | -2.4 (2.5) | **-3.9;-0.9** | -1.7 (2.8) | -3.3;0 | -1.3 (2.8) | -2.7;0.1 | -1.6 (2.4) | **-2.8;-0.4** | -1 (1.7) | -1.9;0 |
| Brainstem | -2.7 (1.3) | **-3.4;-1.9** | -5.2 (1.5) | **-6.1;-4.2** | -2.5 (1.3) | **-3.3;-1.7** | -1.7 (1.6) | **-2.5;-0.9** | -2.7 (2.4) | **-4;-1.5** | -1.4 (1.5) | **-2.2;-0.6** |
| **MRI- mean diffusivity** |  |  |  |  |  |  |  |  |  |  |  |  |
| Putamen | 2.2 (2.8) | **0.5;4** | 2.3 (2.2) | **0.8;3.8** | 0.6 (2.2) | -0.9;2.1 | 1.9 (2.6) | **0.7;3.2** | 2.1 (4.7) | -0.4;4.6 | 0.8 (3.7) | -1.3;2.9 |
| Caudate nucleus | 2.7 (3.2) | **0.7;4.6** | 6.6 (8.8) | **-0.1;13.3** | 3.5 (9.2) | -3.5;10.6 | 2.8 (5.8) | **0;5.6** | 4.7 (7.5) | **0.8;8.5** | 2.5 (6) | -0.7;5.6 |
| Striatum | 2.5 (2.9) | **0.7;4.2** | 4 (5) | -0.2;8.1 | 1.7 (6) | -3.3;6.7 | 2.4 (3.9) | **0.5;4.3** | 3.5 (5.6) | **0.6;6.5** | 1.8 (3.7) | -0.3;3.9 |
| Cerebellum white matter | 1.9 (3.1) | 0;3.7 | 1.3 (6) | -3.3;5.9 | 0.4 (4) | -2.6;3.5 | 2.1 (3.7) | **0.3;3.9** | 0.4 (3.9) | -1.7;2.5 | -0.9 (3.8) | -3.1;1.2 |
| Cerebellum gray matter | 3.6 (1.7) | **2.6;4.6** | 3.4 (4) | **0.5;6.2** | 0 (3.6) | -2.6;2.6 | 1.7 (4.3) | -0.4;3.8 | 1.8 (4.5) | -0.5;4.2 | 0.6 (3.7) | -1.4;2.6 |
| Medulla | 0.2 (6.4) | -4.1;4.5 | 0 (8.2) | -6.3;6.3 | -0.2 (7.7) | -6.1;5.7 | 3.4 (7) | -0.2;7 | 3.3 (7.5) | -0.9;7.5 | 0.9 (4.5) | -1.7;3.5 |
| Pons | 0.7 (3.6) | -1.5;2.9 | 2.9 (7.1) | -1.9;7.6 | 2.4 (5.8) | -1.5;6.2 | 1.7 (7) | -1.8;5.2 | 0.3 (6.1) | -2.9;3.6 | 0.8 (4.5) | -1.8;3.4 |
| Midbrain | 0.5 (3.6) | -1.6;2.7 | 3 (5.1) | -0.4;6.5 | 2.7 (4.3) | -0.2;5.6 | 1 (4.6) | -1.2;3.2 | 2 (4) | **0;4.1** | 1.3 (3.6) | -0.6;3.2 |
| Brainstem | 0 (3.8) | -2.3;2.3 | 1.2 (4.9) | -1.9;4.3 | 1.5 (5.1) | -1.8;4.7 | 2.1 (5.5) | -0.5;4.7 | 1.1 (4.6) | -1.4;3.6 | 0.2 (4.4) | -2.2;2.7 |
| **MRI- fractional anisotropy** |  |  |  |  |  |  |  |  |  |  |  |  |
| Total white matter | -0.9 (1.6) | -1.8;0.1 | 3.2 (7.9) | -1.8;8.2 | 4.2 (7.6) | -0.6;9 | -2.3 (3.4) | -4;-0.6 | -3.5 (5.9) | -6.6;-0.3 | -1.5 (5.5) | -4.6;1.5 |
| Cerebellar white matter | -3.9 (4.3) | **-6.7;-1.2** | -3 (8) | -9.6;3.7 | 1.1 (5.4) | -3.5;5.6 | -1.1 (4.2) | -3.3;1.1 | -2.6 (5) | -5.2;0.1 | -1.4 (6.4) | -5.3;2.4 |
| **Dat-Scan** |  |  |  |  |  |  |  |  |  |  |  |  |
| caudate nucleus – SBR | -5.4 (12.3) | -12.5;1.7 | 0.4 (11) | -6.6;7.4 | 8.3 (16.3) | -2;18.7 | -14.8 (15.1) | -21.7;-7.9 | -23.1 (23.6) | -34.4;-11.7 | -11.3 (25.5) | -24;1.4 |
| putamen– SBR | -6.9 (16.1) | -16.2;2.3 | -12.2 (14.8) | **-21.6;-2.8** | -4.4 (16.8) | -15.1;6.2 | -10.1 (29.1) | -23.3;3.2 | -23.8 (23.8) | -35.3;-12.3 | -16.1 (19.5) | -25.8;-6.4 |
| Striatum | -6.6 (10.6) | **-12.8;-0.5** | -5.4 (10.1) | -11.8;1 | 2.6 (13.4) | -5.9;11.1 | -15.4 (11) | -20.4;-10.4 | -24.7 (19) | -33.8;-15.5 | -13.2 (19.7) | -23.1;-3.4 |
| Putamen vs. caudate ratio | 0.1 (23.2) | -13.3;13.4 | -12.1 (15.6) | **-22;-2.1** | -10.8 (16.2) | **-21.1;-0.5** | 12 (53.6) | -12.4;36.4 | 13.3 (79.7) | -25.1;51.7 | 2.6 (43.8) | -19.2;24.4 |
| **Wet biomarkers** |  |  |  |  |  |  |  |  |  |  |  |  |
| Plasma Neurofilament Light chain | 7.8 (21.8) | -4.8;20.4 | 13.1 (53.1) | -19;45.2 | 2.1 (37.6) | -20.6;24.7 | 9.7 (24.1) | -1.6;20.9 | 22.9 (32.8) | 7.5;38.2 | 14.8 (24.7) | 2.5;27.1 |

**Table S3.** **Biomarkers progression (percentage changes) for MSA-P and MSA-P patients, separately.**

|  |  |  |  |  |  |  | **MSA C vs. MSA P** | | | |
| --- | --- | --- | --- | --- | --- | --- | --- | --- | --- | --- |
|  | **MSA C** | | | **MSA P** | | | **M0-M6** | **M0-M12** | **M6-M12** | **M0-M6-M12** |
| **Characteristics** | **M0-M6** | **M0-M12** | **M6-M12** | **M0-M6** | **M0-M12** | **M6-M12** | **MWW** | **MWW** | **MWW** | **Adjusted model** |
| **Clinical** |  |  |  |  |  |  |  |  |  |  |
| UMSARS |  |  |  |  |  |  |  |  |  |  |
| *I* | 23.9 (25.4) | 33 (23.7) | 11.2 (16.3) | 21.9 (21.2) | 35.9 (20.8) | 17.1 (18.8) | 0,8021 | 0,4618 | 0,3740 | 0,5923 |
| *II* | 16.3 (19.2) | 23.9 (15.8) | 7 (15.9) | 20.0 (21.9) | 32.4 (28.6) | 13.5 (25.8) | 0,6284 | 0,5506 | 0,6896 | 0,2797 |
| *I+II* | 18.8 (19.4) | 27.4 (14.8) | 9 (12.6) | 20.5 (19.2) | 33.3 (19.2) | 14.5 (16.6) | 0,8542 | 0,6991 | 0,3642 | 0,2593 |
| *IV* | 8.3 (36.8) | 26.9 (43.9) | 20.5 (31.3) | 39.7 (73.5) | 63.3 (67.2) | 26.7 (34.9) | 0,1598 | 0,0821 | 0,5929 | 0,1131 |
| MSA-Qol, total | 22.5 (45.5) | 29.4 (36.3) | 12.5 (33.3) | 20.8 (41.1) | 32.9 (36.6) | 13.5 (32) | 0,8281 | 0,7518 | 0,9696 | 0,9443 |
| *Motor* | 25.2 (36.7) | 43.1 (32) | 25.2 (59.1) | 33.7 (55.2) | 56.9 (65.7) | 20 (34.2) | 0,9069 | 0,9439 | 0,7322 | 0,9589 |
| *Non-motor* | 28.5 (48.3) | 28.3 (36) | 8.4 (37.6) | 16.5 (44.5) | 20.4 (30.7) | 19.3 (60.7) | 0,4944 | 0,5394 | 0,6484 | 0,9792 |
| E*motional* | 40.4 (112.2) | 47.2 (122.7) | 5.9 (31.7) | 24.1 (63.4) | 29.3 (63.6) | 10.9 (63.6) | 1,0000 | 0,9160 | 0,5177 | 0,9358 |
| **MRI *- regional atrophy*** |  |  |  |  |  |  |  |  |  |  |
| Putamen | -1.6 (2.9) | -3.2 (3.4) | -1.4 (2.7) | -1.5 (2.3) | -3.5 (4.3) | -1.9 (3.2) | 0,8708 | 0,5906 | 0,2550 | 0,7625 |
| Caudate nucleus | -1.5 (1.7) | -1.2 (2) | 0.2 (2.9) | -2.2 (3.5) | -4.2 (5.7) | -2.2 (3.7) | 0,7865 | 0,0697 | 0,0795 | 0,1044 |
| Total white matter | 0.1 (1.1) | -0.7 (1.3) | -0.9 (1) | -1.1 (1.4) | -1.4 (1.3) | -0.6 (1.4) | 0,0179 | 0,2193 | 0,5023 | 0,0542 |
| Total gray matter | -0.6 (1.3) | -1.4 (1.4) | -0.7 (1.2) | -1.2 (2.1) | -1.7 (1.9) | -0.7 (1.1) | 0,3416 | 0,5351 | 0,9827 | 0,5423 |
| Mean Striatum | -1.6 (2) | -2.3 (2.5) | -0.7 (2.6) | -1.8 (2.4) | -3.9 (4.1) | -2.1 (2.7) | 0,9568 | 0,2347 | 0,1308 | 0,3997 |
| Cerebellum white matter | -4.3 (4.8) | -9.5 (5.2) | -5.4 (5.1) | -4.4 (4.1) | -5 (4.9) | -1.6 (3.3) | 0,8951 | **0,0315** | 0,0836 | 0,3402 |
| Cerebellum gray matter | -1.1 (2.6) | -4.4 (2.6) | -3.1 (1.4) | -2 (1.6) | -2.4 (1.9) | -0.5 (1.9) | 0,2452 | **0,0345** | **0,0026** | **0,0044** |
| Medulla | -1 (2.3) | -1.2 (1.4) | -0.2 (2) | -0.5 (4.3) | -1.8 (3.2) | -0.7 (3.1) | 0,9568 | 0,9339 | 0,9480 | 0,7804 |
| Pons | -4.6 (1.7) | -8.7 (1.8) | -4.2 (1.4) | -2.2 (2.2) | -3.6 (3.2) | -1.9 (1.9) | **0,0089** | **0,0006** | **0,0056** | **0,0134** |
| Midbrain | -0.7 (2.7) | -2.4 (2.5) | -1.7 (2.8) | -1.3 (2.8) | -1.6 (2.4) | -1 (1.7) | 0,8143 | 0,1654 | 0,6180 | 0,6209 |
| Brainstem | -2.7 (1.3) | -5.2 (1.5) | -2.5 (1.3) | -1.7 (1.6) | -2.7 (2.4) | -1.4 (1.5) | 0,0478 | **0,0059** | 0,0667 | 0,0980 |
| **MRI- mean diffusivity** |  |  |  |  |  |  |  |  |  |  |
| Putamen | 2.2 (2.8) | 2.3 (2.2) | 0.6 (2.2) | 1.9 (2.6) | 2.1 (4.7) | 0.8 (3.7) | 0,6759 | 0,5427 | 0,9590 | 0,9904 |
| Caudate nucleus | 2.7 (3.2) | 6.6 (8.8) | 3.5 (9.2) | 2.8 (5.8) | 4.7 (7.5) | 2.5 (6) | 0,6214 | 0,5947 | 0,8446 | 0,8469 |
| Striatum | 2.5 (2.9) | 4 (5) | 1.7 (6) | 2.4 (3.9) | 3.5 (5.6) | 1.8 (3.7) | 0,5691 | 0,6943 | 0,5050 | 0,9931 |
| Cerebellum white matter | 1.9 (3.1) | 1.3 (6) | 0.4 (4) | 2.1 (3.7) | 0.4 (3.9) | -0.9 (3.8) | 1,0000 | 0,3054 | 0,3395 | 0,5667 |
| Cerebellum gray matter | 3.6 (1.7) | 3.4 (4) | 0 (3.6) | 1.7 (4.3) | 1.8 (4.5) | 0.6 (3.7) | 0,1091 | 0,4434 | 0,5849 | 0,1166 |
| Medulla | 0.2 (6.4) | 0 (8.2) | -0.2 (7.7) | 3.4 (7) | 3.3 (7.5) | 0.9 (4.5) | 0,2889 | 0,2943 | 0,9752 | 0,5126 |
| Pons | 0.7 (3.6) | 2.9 (7.1) | 2.4 (5.8) | 1.7 (7) | 0.3 (6.1) | 0.8 (4.5) | 0,6772 | 0,1444 | 0,3213 | 0,2971 |
| Midbrain | 0.5 (3.6) | 3 (5.1) | 2.7 (4.3) | 1 (4.6) | 2 (4) | 1.3 (3.6) | 0,8790 | 0,4309 | 0,4807 | 0,5750 |
| Brainstem | 0 (3.8) | 1.2 (4.9) | 1.5 (5.1) | 2.1 (5.5) | 1.1 (4.6) | 0.2 (4.4) | 0,4265 | 0,6962 | 0,4855 | 0,3799 |
| **MRI- fractional anisotropy** |  |  |  |  |  |  |  |  |  |  |
| Total white matter | -0.9 (1.6) | 3.2 (7.9) | 4.2 (7.6) | -2.3 (3.4) | -3.5 (5.9) | -1.5 (5.5) | 0,1154 | **0,0217** | 0,1142 | **0,0153** |
| Cerebellar white matter | -3.9 (4.3) | -3 (8) | 1.1 (5.4) | -1.1 (4.2) | -2.6 (5) | -1.4 (6.4) | 0,1552 | 0,6943 | 0,3062 | 0,4184 |
| **Dat-Scan** |  |  |  |  |  |  |  |  |  |  |
| caudate nucleus – SBR | -5.4 (12.3) | 0.4 (11) | 8.3 (16.3) | -14.8 (15.1) | -23.1 (23.6) | -11.3 (25.5) | 0,0612 | **0,0085** | **0,0375** | **0,0064** |
| putamen– SBR | -6.9 (16.1) | -12.2 (14.8) | -4.4 (16.8) | -10.1 (29.1) | -23.8 (23.8) | -16.1 (19.5) | 0,3440 | 0,1844 | 0,2068 | 0,5896 |
| Striatum | -6.6 (10.6) | -5.4 (10.1) | 2.6 (13.4) | -15.4 (11) | -24.7 (19) | -13.2 (19.7) | 0,0656 | **0,0057** | **0,0450** | 0,1612 |
| Putamen vs. caudate ratio | 0.1 (23.2) | -12.1 (15.6) | -10.8 (16.2) | 12 (53.6) | 13.3 (79.7) | 2.6 (43.8) | 0,9600 | 0,6733 | 0,7851 | 0,2471 |
| **Wet biomarkers** |  |  |  |  |  |  |  |  |  |  |
| Plasma Neurofilament Light chain | 7.8 (21.8) | 13.1 (53.1) | 2.1 (37.6) | 9.7 (24.1) | 22.9 (32.8) | 14.8 (24.7) | 0,9308 | 0,1767 | 0,0725 | 0,6546 |

MWW = p-value of the Mann-Whitney-Wilcoxon test

Adjusted model = p-value of the interaction of the mixed linear regression model for repeated measures: response variable was the crude values and explanatory variables were the group (MSA-P / MSA-C), the visit (M0, M6, M12) and the interaction group * visit. Each model was adjusted for age and sex.

**Table S4. CSF NfL values.**

| **Biomarkers** | **Statistics** | **MSA Patients at M0** | **MSA Patients at M6** | **MSA Patients at M12** |
| --- | --- | --- | --- | --- |
|  | | | | |
| Neurofilament Light Protein (pg/mL) (Cerebrospinal fluid Origin) | n (nmiss) | 7 (1) | 6 (0) | 5 (0) |
|  | Mean (SD) | 2787.7 (1774.4) | 3557 (2615.4) | 2924.4 (1544.3) |
|  | 95% CI | 1146.7;4428.7 | 812.3;6301.7 | 1006.9;4841.9 |
|  | Coefficient of variation | 63.6 | 73.5 | 52.8 |
|  | Median (Q1;Q3) | 2066 (1090;4260) | 2821 (1726;4740) | 2276 (1942;3960) |
|  | Min ; Max | (736;5520) | (1094;8140) | (1364;5080) |

**Table S5. Cross-sectional correlations at baseline for MSA-(P+C) patients**

| **var1** | **var2** | **n** | **corr** | **l95** | **u95** | **pvalue** |
| --- | --- | --- | --- | --- | --- | --- |
| UMSSC_sum12_M0 | Mean Putamen at M0 (RA - MRI) | 40 | -0.58087 | -0.75565 | -0.32886 | 0.00009 |
| UMSSC_sum12_M0 | Mean Caudate nucleus at M0 (RA - MRI) | 40 | -0.40160 | -0.63382 | -0.10297 | 0.01021 |
| UMSSC_sum12_M0 | Total white matter at M0 (RA - MRI) | 40 | -0.05819 | -0.36312 | 0.25800 | 0.72135 |
| UMSSC_sum12_M0 | Total gray matter at M0 (RA - MRI) | 40 | -0.00235 | -0.31364 | 0.30939 | 0.98852 |
| UMSSC_sum12_M0 | Striatum at M0 (RA - MRI) | 40 | -0.53556 | -0.72596 | -0.26891 | 0.00037 |
| UMSSC_sum12_M0 | Cerebellum white matter (right-left mean) at M0 (RA - MRI) | 40 | 0.08799 | -0.22982 | 0.38885 | 0.58926 |
| UMSSC_sum12_M0 | Cerebellum gray matter (right-left mean) at M0 (RA - MRI) | 40 | 0.13189 | -0.18732 | 0.42591 | 0.41722 |
| UMSSC_sum12_M0 | Medulla at M0 (RA - MRI) | 40 | -0.15201 | -0.44257 | 0.16743 | 0.34909 |
| UMSSC_sum12_M0 | PONS at M0 (RA - MRI) | 40 | 0.11544 | -0.20339 | 0.41213 | 0.47812 |
| UMSSC_sum12_M0 | Midbrain at M0 (RA - MRI) | 40 | -0.00949 | -0.32006 | 0.30292 | 0.95363 |
| UMSSC_sum12_M0 | Whole brainstem at M0 (RA - MRI) | 40 | 0.07182 | -0.24518 | 0.37495 | 0.65964 |
| UMSSC_sum12_M0 | Mean Putamen at M0 (MD - MRI) | 38 | 0.35412 | 0.03882 | 0.60528 | 0.02917 |
| UMSSC_sum12_M0 | Mean Caudate nucleus at M0 (MD - MRI) | 38 | 0.08982 | -0.23667 | 0.39808 | 0.59178 |
| UMSSC_sum12_M0 | Striatum at M0 (MD - MRI) | 38 | 0.28963 | -0.03312 | 0.55768 | 0.07777 |
| UMSSC_sum12_M0 | Cerebellum white matter (right-left mean) at M0 (MD - MRI) | 38 | -0.15452 | -0.45189 | 0.17375 | 0.35429 |
| UMSSC_sum12_M0 | Cerebellum gray matter (right-left mean) at M0 (MD - MRI) | 38 | -0.09409 | -0.40170 | 0.23259 | 0.57418 |
| UMSSC_sum12_M0 | Medulla at M0 (MD - MRI) | 36 | -0.08077 | -0.39873 | 0.25452 | 0.63956 |
| UMSSC_sum12_M0 | PONS at M0 (MD - MRI) | 38 | -0.18918 | -0.47985 | 0.13891 | 0.25532 |
| UMSSC_sum12_M0 | Midbrain at M0 (MD - MRI) | 38 | -0.00614 | -0.32519 | 0.31416 | 0.97081 |
| UMSSC_sum12_M0 | Whole brainstem at M0 (MD - MRI) | 38 | -0.16922 | -0.46381 | 0.15908 | 0.30981 |
| UMSSC_sum12_M0 | Total white matter at M0 (FA - MRI) | 37 | 0.00131 | -0.32285 | 0.32519 | 0.99387 |
| UMSSC_sum12_M0 | Mean Cerebellum white matter at M0 (FA - MRI) | 36 | 0.12237 | -0.21480 | 0.43349 | 0.47708 |
| UMSSC_sum12_M0 | Striatum at M0 (SBR - DatScan) | 41 | -0.56094 | -0.74076 | -0.30612 | 0.00014 |
| UMSSC_sum12_M0 | Plasma Neurofilament Light Protein at M0 | 41 | 0.52481 | 0.25897 | 0.71674 | 0.00043 |

Spearman Correlation matrix for total UMSARS I+II - At M0 - MSA- (P+C) Patients.

**Table S6. Baseline clinical, neuroimaging, and wet biomarkers comparisons for slow vs. fast progressors and dropout (missing patients at M12) vs. all the others.**

|  | **UMSARS I+II** |  |  |  |  |  |
| --- | --- | --- | --- | --- | --- | --- |
|  | **Crude Delta M0->M12** | | | **Test** | **Test** | **Test** |
| **Characteristics at baseline** | **dropout** | **<10pts** | **>=10 pts** | **< vs. >= 10 pts** | **droupout vs. Others** | **droupout vs. <10 pts** |
| **TOTAL** | 7 | 14 | 20 | **p-value** | **p-value** | **p-value** |
| **Age (years)** | 65 (8.9) | 63.1 (9.3) | 62.2 (5.7) | 0.8076 | 0.4599 | 0.659 |
| **Sex (Female)^a^** | 5 (71.4) | 7 (50.0) | 9 (45.0) | 0.7738 | 0.4099^b^ | 0.6424 ^b^ |
| **BMI (Kg/m^2^)** | 25.3 (4.7) | 26.1 (5.9) | 25.9 (4.5) | 0.8993 | 0.9718 | 1,0000 |
| **MSA-P (N, %)** | 5 (71.4) | 8 (57.1) | 13 (65.0) | 0.6427 | 1,0000 ^b^ | 0.6557 ^b^ |
| **MSA-QoL** |  |  |  |  |  |  |
| Total Score | 50.1 (12.2) | 31.2 (11.8) | 39.8 (16.8) | 0.0925 | **0.0275** | **0.0111** |
| Motor sub-score | 57.8 (15.5) | 34.6 (12.9) | 40.7 (16.2) | 0.2628 | **0.0114** | **0.018** |
| Non-motor sub-score | 44.4 (11.9) | 34.2 (13.7) | 37.9 (16.8) | 0.7022 | 0.178 | 0.2309 |
| Emotional sub-score | 47.2 (21.7) | 25.1 (18) | 40.4 (24) | 0.0833 | 0.1484 | 0.0228 |
| **Moca** | 27.1 (2.3) | 27.1 (3) | 27 (2.2) | 0.7128 | 0.917 | 1,0000 |
| **BDI** | 22.3 (8.4) | 10.3 (3.2) | 16.2 (7.6) | **0.0292** | **0.0166** | **0.003** |
| **Compass-31** |  |  |  |  |  |  |
| Orthostatic intolerance sub-score | 19.4 (14.3) | 16.9 (14.4) | 16.6 (13.1) | 0.9717 | 0.5987 | 0.6509 |
| Vasomotor sub-score | 0 (0) | 0.7 (1.5) | 0.2 (0.6) | 0.314 | 0.3063 | 0.2347 |
| Secretomotor sub-score | 6.4 (2.8) |  | 3.9 (3.9) | 0.0707 | 0.3055 | 0.9396 |
| Gastrointestinal sub-score | 7.1 (3.2) | 6.7 (3.4) | 6.5 (3.5) | 0.9305 | 0.717 | 0.8242 |
| Bladder sub-score | 4.4 (2.6) | 4.1 (2.4) | 4.1 (2.9) | 0.8742 | 0.7672 | 0.8224 |
| Pupillomotor sub-score | 2.1 (0.9) | 1.8 (1.2) | 1.9 (1.2) | 1 | 0.5453 | 0.5532 |
| Total score | 39.6 (15.4) | 36.5 (17.6) | 33.2 (17.1) | 0.6397 | 0.3464 | 0.633 |
| **MRI *- regional atrophy*** |  |  |  |  |  |  |
| Putamen | 3160.3 (1297.5) | 4016.7 (851.3) | 3785.2 (1112) | 0.6781 | 0.1023 | 0.0825 |
| Caudate nucleus | 3196.8 (712.8) | 3373.3 (512.1) | 3195 (572.8) | 0.4607 | 0.621 | 0.4867 |
| Total white matter | 464240 (62373.2) | 442930.7 (42376.7) | 440098.3 (51289.9) | 0.928 | 0.5723 | 0.5332 |
| Total gray matter | 596497.5 (44805.4) | 583029.5 (40454.6) | 575878.8 (52236.3) | 0.7865 | 0.4592 | 0.633 |
| Mean Striatum | 6357.1 (1999.4) | 7390 (1289) | 6980.2 (1651.5) | 0.652 | 0.1625 | 0.1088 |
| Cerebellum white matter | 11790.5 (3977.6) | 10545.5 (3344) | 10334 (3171.6) | 0.7865 | 0.3604 | 0.4426 |
| Cerebellum gray matter | 47950.3 (7287.1) | 46735.6 (5250.4) | 46429 (7006.1) | 0.5284 | 0.5964 | 0.5332 |
| Medulla | 4430.8 (453.7) | 4867.2 (394.7) | 4769 (497.3) | 0.6263 | 0.0574 | 0.062 |
| Pons | 12775 (2901.3) | 12500.3 (2976.8) | 12717 (3397.5) | 0.8425 | 0.9436 | 1 |
| Midbrain | 5778.9 (762) | 5781.6 (565.2) | 5786.2 (727.9) | 0.9568 | 0.8045 | 0.7406 |
| Brainstem | 23194.6 (3846.1) | 23372 (3642.8) | 23490.4 (4363.5) | 0.9568 | 0.7773 | 0.7406 |
| **MRI- mean diffusivity** |  |  |  |  |  |  |
| Putamen | 0.8 (0.1) | 0.8 (0.1) | 0.8 (0.1) | 0.9525 | 0.4127 | 0.4379 |
| Caudate nucleus | 1 (0.1) | 1 (0.1) | 1 (0.1) | 0.3749 | 0.085 | 0.3943 |
| Striatum | 1.8 (0.1) | 1.8 (0.2) | 1.7 (0.2) | 0.5659 | 0.1719 | 0.3536 |
| Cerebellum white matter | 0.8 (0.1) | 0.8 (0.1) | 0.9 (0.1) | 0.9842 | 0.2359 | 0.1938 |
| Cerebellum gray matter | 1 (0.1) | 1 (0.2) | 1 (0.2) | 0.9209 | 1 | 1 |
| Medulla | 1 (0.1) | 1 (0.1) | 1.1 (0.2) | 0.6146 | 0.2879 | 0.5341 |
| Pons | 0.9 (0.1) | 1 (0.2) | 1 (0.2) | 0.4183 | 0.0534 | 0.0725 |
| Midbrain | 1 (0.1) | 1 (0.1) | 0.9 (0.1) | 0.1337 | 0.7087 | 0.8757 |
| Brainstem | 0.9 (0.1) | 1 (0.1) | 1 (0.1) | 0.3962 | 0.1311 | 0.1295 |
| **MRI- fractional anisotropy** |  |  |  |  |  |  |
| Cerebellar white matter | 0.3 (0) | 0.4 (0.1) | 0.3 (0.1) | 0.9475 | 0.9683 | 0.3943 |
| Total white matter | 0.3 (0) | 0.3 (0) | 0.3 (0) | 0.3867 | 0.6863 | 0.9005 |
| **Dat-Scan** |  |  |  |  |  |  |
| caudate nucleus – SBR | 1.9 (1.3) | 3.1 (1.3) | 2.8 (1.2) | 0.3975 | 0.0573 | 0.0949 |
| putamen– SBR | 1 (1.2) | 1.9 (1.1) | 1.4 (1) | 0.1248 | 0.0764 | 0.0535 |
| Putamen vs. caudate ratio | 0.5 (0.2) | 0.6 (0.1) | 0.5 (0.2) | 0.0807 | 0.823 | 0.2612 |
| Striatum | 1.4 (1.2) | 2.5 (1.2) | 2 (1) | 0.2495 | 0.0663 | 0.062 |
| **Wet biomarkers** |  |  |  |  |  |  |
| Plasma Neurofilament Light chain | 65.5 (33.6) | 29.6 (7.1) | 36.8 (13.9) | 0.0894 | **0.0125** | **0.0058** |

^a^n (%) and Chi2 Test; ^b^Fisher Test; fast progressor: patients with a increment ≥10 points at the total UMSARS I+II from M0 to M12.

**Table S6**. Clinical and biomarkers details of the 5 MSA-C patients with normal Dat-Scan

| *Pt code* | *Disease duration at baseline (months)* | *Dysautonomia criteria & supportive criteria* | *MRI signatures* | *Plasma NfL, pg/ml* | *Sleep disorders* | *Negative Genetic tests* |
| --- | --- | --- | --- | --- | --- | --- |
| *01-014* | *36* | *Neurogenic OH and PVR> 100 ml*  *Early Dysarthria* | *Pons and cerebellar atrophy* | *49.4* | *RBD, nocturnal stridor* | *FXTAS, SCA 1,2,3,6,7,12,17* |
| *02-009* | *48* | *Neurogenic OH, urinary urgency with episodic incontinence* | *Hot-cross bun sign, pons and cerebellar atrophy* | *25.4* | *RBD* | *NA* |
| *04-001* | *53* | *Neurogenic OH, urinary incontinence, early dysphagia and dysarthria* | *Cerebellar atrophy* | *25.5* | *RBD* | *Friedrich ataxia and Niemann Pick type C* |
| *06-002* | *13* | *Neurogenic OH, PVR> 100 ml, Erectile dysfunction < 60 years* | *Marked pons and middle cerebellar*  *peduncle* | *20.5* | *NA* | *NA* |
| *08-004* | *27* | *Urinary urgency with episodic incontinence Erectile dysfunction < 60 years, Unexplained Babinski sign* | *Hot-cross bun sign* | *27.5* | *RBD* | *NA* |
